# Identification and Functional Characterization of a Soybean (*Glycine max*) Thioesterase that Acts on Intermediates of Fatty Acid Biosynthesis

**DOI:** 10.3390/plants8100397

**Published:** 2019-10-08

**Authors:** Huong Thi Diem Tran, Nhan Trong Le, Vy Le Uyen Khuat, Thuong Thi Hong Nguyen

**Affiliations:** Faculty of Biology and Biotechnology, University of Science, Vietnam National University HCMC, Ho Chi Minh 700000, Vietnam; ttdhuong93@gmail.com (H.T.D.T.); ltnhan17@gmail.com (N.T.L.); kluvy@hcmus.edu.vn (V.L.U.K.)

**Keywords:** 2-methylketones, 3-hydroxyacids, 3-ketoacids, acyl-ACP thioesterase, *Glycine max*, methylketone synthase 2 (MKS2)

## Abstract

(1) Background: Plants possess many acyl-acyl carrier protein (acyl-ACP) thioesterases (TEs) with unique specificity. One such TE is methylketone synthase 2 (MKS2), an enzyme with a single-hotdog-fold structure found in several tomato species that hydrolyzes 3-ketoacyl-ACPs to give free 3-ketoacids. (2) Methods: In this study, we identified and characterized a tomato MKS2 homolog gene, namely, *GmMKS2*, in the genome of soybean (*Glycine max)*. (3) Results: *GmMKS2* underwent alternative splicing to produce three alternative transcripts, but only one encodes a protein with thioesterase activity when recombinantly expressed in *Escherichia coli*. Heterologous expression of the main transcript of *GmMKS2*, *GmMKS2-X2*, in *E. coli* generated various types of fatty acids, including 3-ketoacids—with 3-ketotetradecenoic acid (14:1) being the most abundant—*cis*-Δ5-dodecanoic acid, and 3-hydroxyacids, suggesting that GmMKS2 acts as an acyl-ACP thioesterase. In plants, the *GmMKS2-X2* transcript level was found to be higher in the roots compared to other examined organs. *In silico* analysis revealed that there is a substantial enrichment of putative *cis*-regulatory elements related to disease-resistance responses and abiotic stress responses in the promoter of this gene. (4) Conclusions: GmMKS2 showed broad substrate specificities toward a wide range of acyl-ACPs that varied in terms of chain length, oxidation state, and saturation degree. Our results suggest that GmMKS2 might have a stress-related physiological function in *G. max*.

## 1. Introduction

In plants, the formation of acyl carrier protein (ACP) thioesters of acyl groups containing between 2 and 16 carbon atoms occurs in fatty acid (FA) biosynthesis and is catalyzed by a set of fatty acid synthase (FAS) enzymes highly conserved across various species [1,2]. However, plants contain general and lineage-specific acyl-ACP thioesterases (acyl-ACP TEs) that hydrolyze acyl-ACPs of specific length and structure to generate free fatty acids that are used in further metabolism [3,4]. Plants also have thioesterases (TEs) that use fatty acyl-CoA substrates (which occur, for example, during fatty acid degradation) that similarly serve to generate free fatty acids.

All known fatty acyl-TEs have been grouped into 23 different families according to their amino acid sequence, but on the basis of the tertiary structure, they mainly fall into two types of folds: the α/β-hydrolase fold superfamily and the hotdog fold superfamily [5]. Enzymes in the families TE1–TE19 cleave bonds between acyl groups and CoA or ACP, and those in the families TE20–TE23 break bonds between acyl groups and both proteins and glutathione and its derivatives. Most notably, the TE14 family contains many enzymes with acyl-ACP hydrolase (EC 3.1.2.14) activity, including FatA and FatB—two types of acyl-ACP thioesterases highly conserved in plants. FatA and FatB are double-hotdog-fold thioesterases and act preferentially on oleoyl-ACP and saturated acyl-ACP substrates, respectively, to make fatty acid products for the synthesis of a variety of membrane and storage lipids in plants [6,7,8]. In addition to the well-characterized double-hotdog-fold TEs such as FatA- and FatB-type enzymes, plants also possess single-hotdog-fold acyl-ACP TEs, which have only been discovered within the last 10 years [9,10]. The methylketone synthase 2 from wild tomato (*Solanum habrochaites* subsp. *glabratum*) (*Sh*MKS2) was the first enzyme of this type to be characterized. ShMKS2 is localized in chloroplasts and is involved in the hydrolysis of 12–16C 3-ketoacyl-ACPs, intermediates formed during fatty acid biosynthesis. The released 3-ketoacids are converted to 2-methylketones by a decarboxylase, ShMKS1 [9], and some of these volatile 2-methylketones have insecticidal activity [11]. *Arabidopsis* and other plants also have genes homologous to *ShMKS2*, also known as acyl-lipid thioesterases (ALTs); however, their *in planta* function has not been conclusively identified [9,10,12].

*Glycine max* (L.) Merr, a species of the family Fabaceae, is important worldwide for seed protein and oil content and for its capacity to fix atmospheric nitrogen through symbiotic interaction with soil-borne bacteria. Its seed oil has a high content of unsaturated fatty acids (12% linolenic acid (C 18:3), 48% linoleic acid (C 18:2), and 16% oleic acid (C 18:1)) and a lower content of saturated fatty acids (5% stearic acid (C 18:0) and 17% palmitic acid (C 16:0)) [13]. A correlation between the substrate specificity of acyl-ACP thioesterases and the fatty acid profile of the organism from which the enzyme was sourced has been noted previously [14,15], yet none of the functional thioesterase genes in soybean has been described to date.

The specialized metabolites 2-methylketones, the decarboxylated products of 3-ketoacids generated through hydrolysis of 3-ketoacyl-ACPs by MKS2/ALT-like thioesterases, are valuable compounds with applications as pesticides, fragrance and flavor additives, and feedstocks for biofuel production [16,17,18]. Recently, 2-tridecanone and other medium-chain 2-methylketones have been demonstrated to act as chemical cues that affect bacterial behavior on surfaces and hinder plant–bacteria interactions. Notably, the application of 2-tridecanone interfered not only with the pathogenic interaction between a bacterium and its plant host but also with the symbiotic *Rhizobium*–legume interaction [19]. Searches in the database have revealed that *G. max* possesses only one *MKS2*/*ALT*-like gene, but expression in *Escherichia coli* of a chemically synthesized gene for the GmALT failed to show any thioesterase activity of this protein [12], possibly because an inactive alternative-splicing sequence was used (see below).

Taken together, these observations have led us to examine the possible functional significance of the single-hotdog-fold 3-ketoacyl-ACP thioesterase related to ShMKS2 in *G. max* (GmMKS2).

## 2. Results

### 2.1. Identification of Alternatively Spliced MKS2 Isoforms in G. max

**Figure 1:**
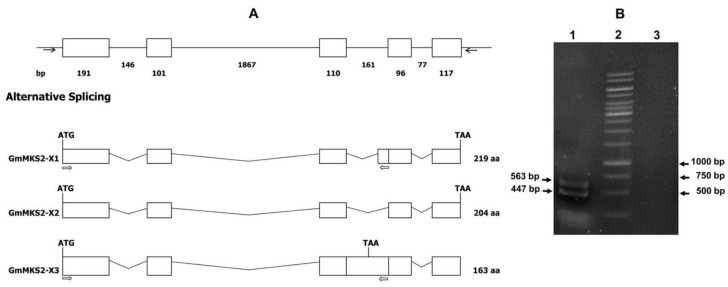
Schematic diagram of the *Glycine max* methylketone synthase 2 (*GmMKS2*) gene and detection of its alternatively spliced products. (**A**) Schematic genomic organization of *GmMKS2* and alternative RNA splicing to generate *GmMKS2-X1*, *-X2*, and *-X3* transcript variants. Exons are represented as boxes, and introns are represented as lines. The sizes (bp) of exons and introns are shown below the boxes and lines. Forward and reverse primers utilized for the amplification of the transcript variants X1, X2, and X3 are indicated by black arrows; (**B**) Detection of *GmMKS2-X1* and *GmMKS2-X3* transcripts using a forward primer located in the first exon and a reverse primer located in the last 45 nucleotides of intron 3, inserted in-frame between exon 3 and 4. Lane 1: two fragments corresponding to the first 447 and 563 nucleotides of *GmMKS2-X1* and *GmMKS2-X3*, respectively; lane 2: DNA ladder; lane 3: negative control.

To identify soybean (*G. max*) genes encoding proteins with homology to methylketone synthase 2 from the wild tomato *S. habrochaites* (ShMKS2), we performed a TBLASTN search to query ShMKS2 (Genbank accession no. ADK38536) against William 82 Assembly 2 (Wm82.a2) Genomic Sequence Database (https://soybase.org). This search identified only one *MKS2*-like gene sequence encoding a protein with homology to ShMKS2, which we named *GmMKS2*. The *GmMKS2* gene is located on chromosome 1. The genomic sequence of the *GmMKS2* gene was obtained by PCR and verified by sequencing (Supplementary File 1). A BLASTN search using the predicted *GmMKS2* genomic sequence as a query against the RefSeq RNA database at NCBI of *G. max* (taxid: 3847) identified two transcript variants potentially transcribed from this locus: variant X1 (accession number XM_006573172.3) and variant X2 (accession number XM_003516775.4). To validate the predicted splice variants, we screened expressed sequence tags (EST) (http://ocri-genomics.org/ocsESTdb/blast/blast.html) and found two representative ESTs in soybean immature seed tissue (Genbank IDs HO025806.1 and HO009286.1), which exhibited over 99.5% nucleotide sequence identity to each other and appeared to correspond to the transcript variant X2 (*GmMKS2-X2*). On the basis of the similarity to ShMKS2 and related sequences from *Arabidopsis thaliana* and the cultivated tomato species, it was predicted that *GmMKS2* consists of five exons and four introns and that *GmMKS2-X2* is the functional transcript of the gene (Figure 1A). The full-length *GmMKS2-X2* transcript was obtained using RNA prepared from immature seeds. This transcript encodes a small protein of 204 amino acids with a predicted molecular mass of 23.1 kDa.

The sequence of the transcript variant X1 (660 bp) is identical to that of the transcript variant X2, except for the insertion of 45 intronic nucleotides between exon 3 and exon 4. To check the existence of transcript variant X1, RT-PCR was performed using the forward primer located at the beginning of the transcript and the reverse primer positioned within the 45-nucleotide insertion region. Analysis of the DNA fragments produced in this experiment by agarose gel electrophoresis gave two sharp bands: one below 500 bp, and the other above 500 bp (Figure 1B). As expected from its size, subsequent sequencing analysis showed that the sequence of the smaller product was 100% identical to the first 447-nucleotide-long sequence of the transcript variant X1. This variant, designated as *GmMKS2-X1*, is produced by using an alternative 3′ acceptor site located in the middle of intron 3, leading to mis-splicing of the latter part of the intron. Sequencing of the amplicon larger than 500 bp revealed an additional transcript variant (designated as GmMKS2-X3) that has not yet been curated in the RefSeq database. Next, we optimized cDNA amount, annealing temperature, cycling time, and annealing/extension time of RT-PCR reactions in order to obtain as many transcript variants of GmMKS2 as possible. As a result, two alternative splicing forms, GmMKS2-X1 and GmMKS2-X3, were simultaneously amplified with the same set of primers for the amplification of the full-length GmMKS2-X2 (Supplementary File 2). As described above, the GmMKS2-X1 variant contains an in-frame insertion of 45 intronic nucleotides that gives rise to a longer GmMKS2 isoform with 15 extra amino acids compared to the GmMKS2-X2 isoform. In the GmMKS2-X3 variant, the entire intron 3 was retained, leading to a premature stop codon within the third exon. This variant was therefore predicted to encode a protein that is shorter by 41 amino acid residues (Supplementary File 3).

### 2.2. Expression of GmMSK2-X2, but Not of the Other Two Variants, in E. coli Generated Predominantly Unsaturated 3-Ketoacids

Like other plant acyl-ACP thioesterases, which are nuclear-encoded and plastid-targeted, GmMKS2 isoforms contain a putative plastid-targeting signal peptide at the N-terminus, with a cleavage site located between Gly^64^ and Met^65^. For the determination of the in vivo activity of the three GmMKS2 isoforms, an open reading frame (ORF) of each GmMKS2 isoform that began at Met-65 was cloned into the pETDuet-1 vector, the resulting vector was introduced into *E. coli* C41(DE3), and its expression was induced in this host strain. As observed by SDS-PAGE analysis, GmMKS2 was strongly expressed in *E. coli* when induced with 0.5 mM IPTG at 18 °C for 16 h. Under this condition, protein analysis by SDS-PAGE showed that the GmMKS2-X1, GmMKS2-X2, and GmMKS2-X3 isoforms were expressed in both soluble and insoluble forms (Supplementary File 4).

**Figure 2:**
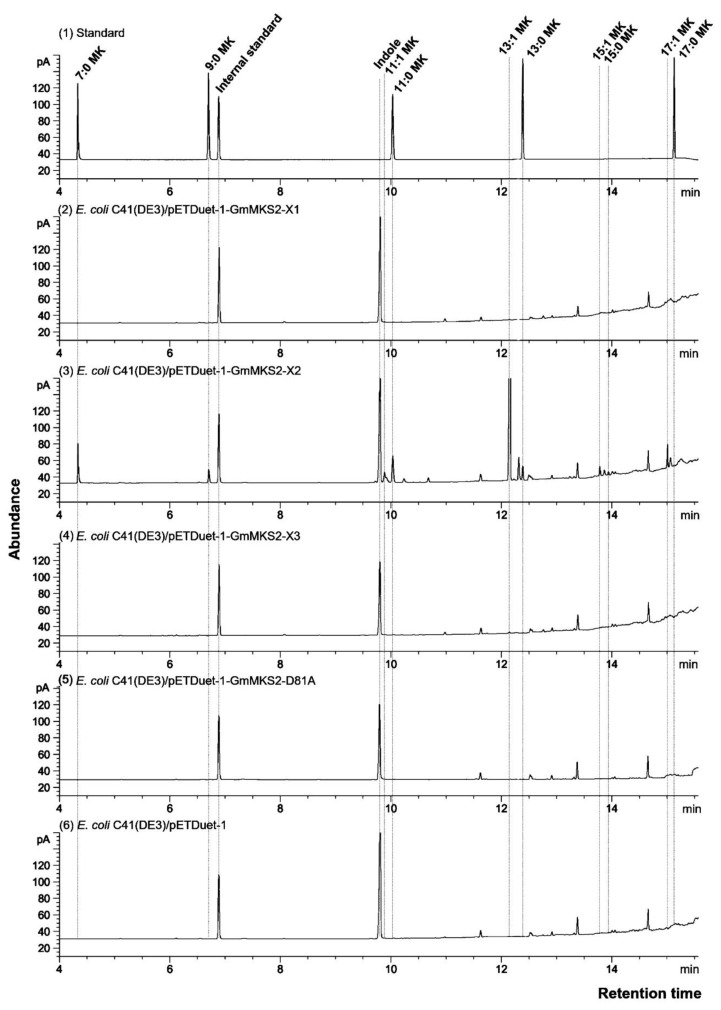
Gas chromatography (GC) analysis of the peaks obtained after heating the culture of *Escherichia coli* C41(DE3) cells. Cells carried an empty vector (pETDuet-1) (**6**) or expressed GmMKS2-X1 (**2**), GmMKS2-X2 (**3**), GmMKS2-X3 (**4**), or GmMKS2-X2-D81A (**5**). Peaks were identified by mass spectrometry (MS), and their retention times were compared to those of authentic standards (**1**). Cells were grown, and cultures were collected and treated as described in “Materials and Methods”. Linalool was added as an internal standard before extraction.

The compounds 3-ketoacids are inherently unstable and decarboxylate spontaneously, particularly when heat is applied, to give 2-methylketones with an odd number of carbons [9,20]. Gas chromatography–mass spectrometry (GC–MS) analysis of the culture of *E. coli* C41 (DE3) expressing GmMKS2-X1, -X2, and -X3 after heat-activated decarboxylation was used to identify the production of 3-ketoacids. This analysis identified the presence of odd-chain 2-methylketones including 7:0, 9:0, 11:1, 11:0, 13:1, 13:0, 15:1, 15:0, and 17:1 methylketones, with 2-tridecenone (13:1) being the most abundant (6022.97 ± 105.14 ng/OD unit) in *E. coli* cells expressing GmMKS2-X2 (Table 1). The compounds were either absent or undetectable in the culture of *E. coli* C41 (DE3) expressing GmMKS2-X1 or GmMKS2-X3 or carrying an empty plasmid pETDuet-1 (Figure 2). These results suggest that the corresponding 3-ketoacids ranging from C8 to C18, which were converted to methylketones for GC–MS analysis, were present in the culture of *E. coli* C41 (DE3) expressing GmMKS2-X2. Previous studies demonstrated that extractions without heat-activated decarboxylation reduced the level of all methylketone types significantly, regardless of their carbon chain length [9].

Alignment of the soybean MKS2-X2 with previously characterized MKS2s from *S. habrochaites, Solanum lycopersicum, A. thaliana,* and *Pseudomonas* sp strain CBS3 revealed the characteristically conserved aspartate residue required for thioesterase activity (Supplementary File 5). The corresponding aspartate residue in the *Pseudomonas* sp. enzyme, 4-hydroxybenzoyl-CoA thioesterase, was identified as an acid residue in the active site participating in hydrolysis via a nucleophilic attack of the carboxylate side chain in the thioester moiety of the substrate [21,22]. Accordingly, substitution of the equivalent Asp residue in *Sh*MKS2 and ALT1 by Ala abolished the thioesterase activity of these enzymes [9,10]. In order to further characterize the consequences of the mutation of the conserved aspartate for the functional protein isoform GmMKS2-X2, we constructed the mutant GmMKS2-X2-D81A (mutating the conserved Asp encoded by codon 81 of the complete open reading frame) by site-directed mutagenesis (Supplementary File 5). As expected, expression of the mutated gene (without the transit peptide encoding region) in the *E. coli* C41(DE3) expression strain generated no detectable levels of 3-ketoacids in the culture (Figure 2).

### 2.3. GmMKS2-X2 Could Also Act on cis-Δ5-dodecenoyl-ACP and 3-hydroxyacyl-ACPs

**Figure 3:**
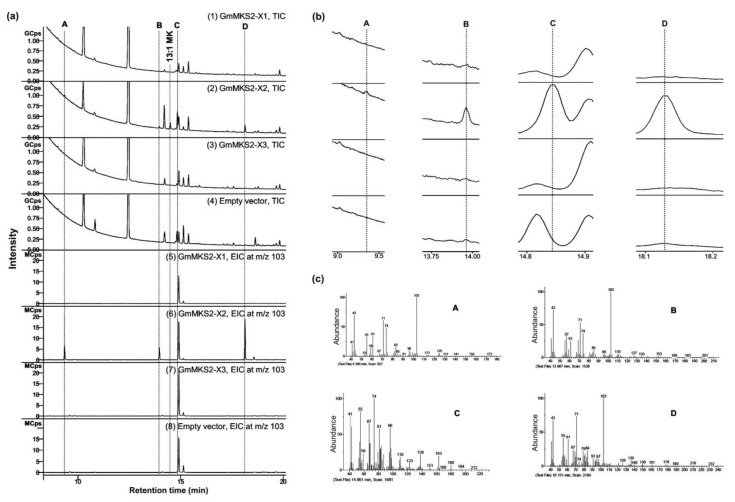
Analysis by GC of methyl esters obtained from fatty acids secreted into the culture by *E. coli* C41(DE3) cells carrying an empty vector or expressing GmMKS2-X1, GmMKS2-X2, or GmMKS2-X3. (**a**) Representative total ion chromatogram (TIC, 1–4) and extracted ion chromatogram at *m*/*z* 103 (EIC at m/z 103, 5–8) of GC–MS analysis for the detection of methyl ester derivatives of 3-hydroxy fatty acids; (**b**) Enlarged image of the labeled peaks; (**c**) Mass spectra of the labeled peaks. The peaks were identified by MS. A: β-hydroxyoctanoic acid (β-OH 8:0 FA), B: 3-hydroxydecanoic acid (β-OH 10:0 FA), C: methyl *cis*-Δ5-dodecenoate, and D: β-hydroxydodecanoic acid (β-OH 12:0 FA). Cells were grown, and cultures were collected and treated as described in “Materials and Methods”.

To further look into the substrate specificities of GmMKS2, fatty acids secreted into the culture medium were methyl-esterified and analyzed via GC–MS (Figure 3). *E. coli* harboring an empty pETDuet-1 vector was used as a negative control. Bacteria expressing the GmMKS2-X2 were found to produce *cis*-Δ5-dodecenoic acid (Figure 3, peak C) and several types of 3-hydroxyacids (Figure 3, peaks A, B, and D) from the endogenous acyl-ACP substrate pool. The compounds eluted at 9.360, 13.967, and 18.151 min showed highly similar mass spectral fragmentation patterns, respectively, to β-hydroxyoctanoic acid (β-OH 8:0 FA), 3-hydroxydecanoic acid (β-OH 10:0 FA), and β-hydroxydodecanoic acid (β-OH 12:0 FA), with the presence of the base peak at *m*/*z* 103 derived from an alpha-cleavage in correspondence of the carbon carrying the hydroxyl group. Again, GmMKS2-X1 and GmMKS2-X3 did not show thioesterase activity toward *cis*-Δ5-dodecenoyl-ACP and 3-hydroxyacyl-ACPs (Figure 3).

### 2.4. Expression Analysis of GmMKS2 in Different Tissues of Soybean Plants

**Figure 4:**
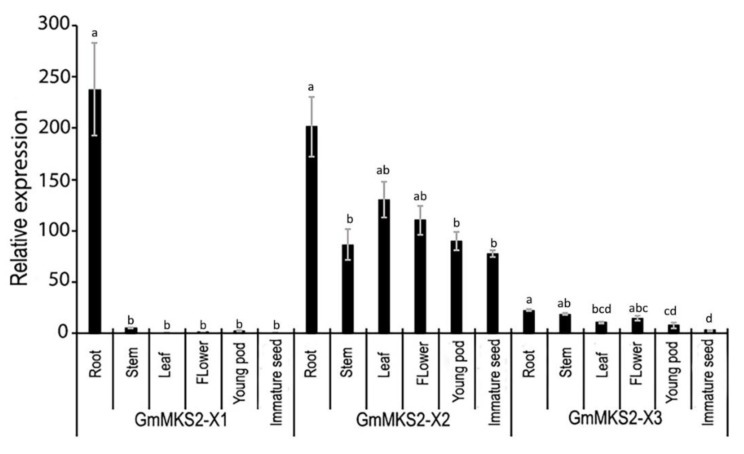
Relative expression levels of *GmMKS2* transcripts in different tissues of *G. max*, determined by qRT-PCR. Expression values were normalized to those of *cons6* (Genbank accession: NM_001254106.2). Each value is the mean ± standard error (SE) from three biological replicates. Significant differences were assessed by one-way ANOVA followed by Tukey’s HSD test. Bars of the same transcript with different letters are significantly different (*p* < 0.01).

The levels of each of the three identified transcripts (*GmMKS2-X1*, *GmMKS2-X2*, and *GmMKS2-X3*) in different tissues of soybean were measured by qRT-PCR (Figure 4). *GmMKS2-X1* was primarily expressed in the roots; *GmMKS2-X2* expression was the highest in the roots and compared to all other tissues examined. *GmMKS2-X3* was also found in all tissues of seven-week-old plants, but at very low levels compared with *GmMKS2-X2* (Figure 4).

### 2.5. Promoter Prediction and Identification of cis-Regulatory Elements in the GmMKS2 Promoter

**Figure 5:**
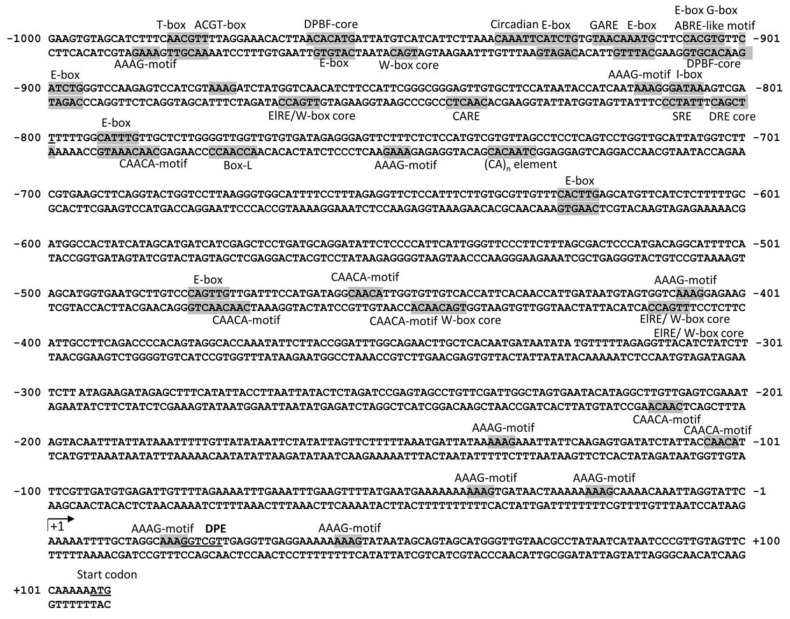
Putative responsive *cis*-acting elements in the *GmMKS2* promoter. The putative promoter region spanning from −1000 to +101 relative to the *GmMKS2* transcription start site was amplified from *G. max* genomic DNA, sequenced, and scanned through PLACE for the identification of number and position of various *cis*-acting regulatory elements. Sequences corresponding to putative regulatory elements are shaded. The arrow indicates the transcription start site.

The 1200-bp sequence of the 5′ flanking sequence of *GmMKS2* was amplified from *G. max* genomic DNA, sequenced, and analyzed to identify a transcription start site (TSS) using TSSPlant, a tool that predicts both TATA and TATA-less promoters in sequences of a wide range of plant genomes [23]. The transcription start site (A_+1_) was predicted to be located at −106 bp upstream of the translation start codon ATG, and a putative promoter region was defined to span from −1000 to +101 bp relative to the transcription start site (Supplementary File 1). The putative core promoter region of *GmMKS2* lacks a TATA box and is, therefore, referred to as a TATA-less promoter [24]. Instead, there is a probable downstream promoter element (DPE) from position +29 to position +34 (GGTCGT) that matches the DPE consensus sequence (RGWYV(T); R = A/G, W = A/T, Y = C/T, V = A/C/G) (Figure 5). *In silico* analysis of the promoter sequence was performed using the software programs NSITE-PL (http://www.softberry.com) and PLACE [25]. This analysis led to the identification of multiple short DNA motifs of 5–10 bases that were predicted to act as phytohormone-responsive *cis*-elements (Supplementary File 6). These include an abscisic acid-responsive element (ABRE)-like sequence (ACGTG) at −909 bp, a gibberellin-responsive element (GARE, TAACAAR) at −922 bp, ten Arabidopsis response regulator (ARR)- binding sites (NGATT) involved in cytokinin responsiveness, and an elicitor responsive element (ElRE) at −867 bp and −414 bp. All these *cis*-regulatory elements, or motifs, are often present in the promoters of genes related to plant stress modulation pathways and play an important role in regulating the expression of various stress responsive genes [26]. Most of these motifs were found to be distributed randomly in both the positive and the negative strands of the promoter sequence of *GmMKS2*.

The *GmMKS2* promoter region also includes potential binding sites for transcription factors involved in specific regulation of transcription, such as basic helix–loop–helix (bHLH) and basic leucine zipper (bZIP) transcription factors and WRKY and MYB proteins (Supplementary File 6). Notably, the E-box consensus sequence (CANNTG), also known as MYC motif, was the most abundant *cis*-regulatory elements found in the *GmMKS2* promoter (Figure 5). In addition to the presence of eight E-boxes, the *GmMKS2* promoter also contains four W-boxes [(T)TGAC(C/T)] with a typical TGAC core sequence required for specific binding of WRKY proteins to the promoter region of target genes [27] (Figure 5). The WRKY transcription factors are key regulators of many plant stress responses, including abiotic stress tolerance and responses to wounding and pathogen attack [28].

## 3. Discussion

### 3.1. Soybean Contains Only a Single Functional Ortholog of the Wild Tomato Methylketone Synthase 2 (ShMKS2)

Database searches have identified multiple homologs of GmMKS2 in a wide range of plant species. There is usually more than one *MKS2*-like gene in each plant genome [10,12,29], but soybean contains only a single homolog gene. The closest homolog (by sequence identity) having both structure and enzymatic activities that has been characterized is 4-hydroxybenzoyl-CoA thioesterase (4HBT) from *Pseudomonas* sp. strain CBS-3. Although the known MKS2/ALT protein sequences from plants share a low level of identity (less than 15%) to 4HBT, they conserve a catalytic Asp residue as the bacterial enzyme (Supplementary File 5) [9,21]. A multiple sequence alignment of GmMKS2-X1 and GmMKS2-X2 with other functionally characterized MKS2s from tomato and *Arabidopsis* plants showed the high sequence similarity among these proteins, especially in the C-terminal region that forms a single hotdog fold (Supplementary File 5). GmMKS2-X3 shares the same its N-terminal 134 amino acids with GmMKS2-X1 and GmMKS2-X2 up to the end of exon 3, then the retention of intron 3 in GmMKS2-X3 gives rise to a different C-terminal sequence. Thus, the last 30 amino acids encoded by intron 3 in GmMKS2-X3 are completely different from the corresponding sequence encoded by exon 4 in GmMKS2-X1, GmMKS2-X2, and other previously reported plant MKS2s. Among the experimentally characterized proteins, the most closely related to GmMKS2-X2 is the *Arabidopsis* acyl-lipid thioesterase 3 (ALT3), with 63.2% of sequence identity between the two proteins. A phylogenetic tree with GmMKS2-X2 and other MKS2s/ALTs identified so far shows that GmMKS2-X2 is clustered with acyl-lipid thioesterases from *A. thaliana* (ALT1-4) on a separate branch diverged from the Solanaceae MKS2 branch (Supplementary File 7).

### 3.2. The Main Isoform of GmMKS2 Has Broad Substrate Specificities toward a Wide Range of Acyl-ACPs That Vary in Terms of Chain Length, Oxidation State, and Saturation Degree

The difficulty of preparing a wide range of 3-ketoacyl-ACP, 3-hydroxyacyl-ACP, and other acyl-ACP substrates precludes an in vitro characterization of acyl-ACP thioesterase. However, the well-known and widely used approach of analyzing fatty acid components produced by thioesterases heterologously expressed in *E. coli* provided an efficient and fast way to study the in vivo activity of these enzymes. In this experimental system, we were not able to observe 3-ketoacids directly but rather assumed their presence on the basis of the detection of 2-methylketones following chemical decarboxylation of the 3-ketoacids [9,30]. GmMKS2-X2, for which the corresponding cDNA was isolated from the immature seeds of *G. max,* produced primarily 2-tridecenone (13:1) and smaller amounts of other odd-chain methylketones (7:0, 9:0, 11:0, 11:1, 13:0, 15:0, 15:1, and 17:1) when expressed in *E. coli* and upon heat-activated decarboxylation. This suggests that their corresponding unsaturated 3-keto fatty acids 14:1, which was converted to unsaturated methylketone 13:1 for GC–MS analysis, were mostly present in the culture medium.

**Figure 6:**
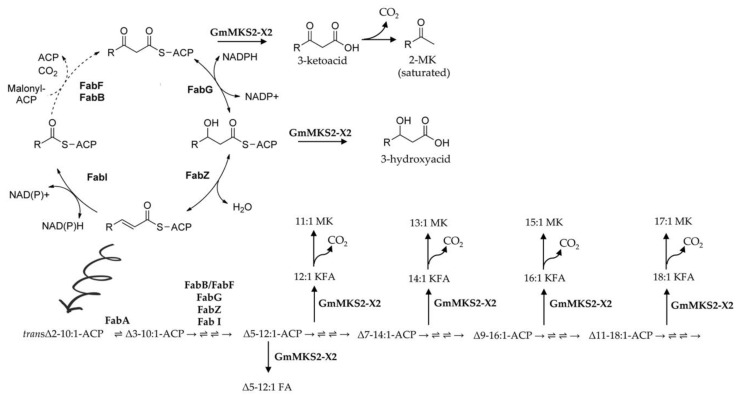
GmMKS2-X2 acts on a broad range of acyl-ACPs, intermediates in fatty acid biosynthesis in *E. coli*. Unsaturated fatty acid biosynthesis in *E. coli* begins with the isomerization of *trans*-2-decenoyl-ACP to *cis*-3-decenoyl-ACP by FabA. This intermediate is then condensed with malonyl-ACP by FabB instead of being reduced. The resulting unsaturated β-ketoacyl-ACP is processed similar to its saturated counterpart, until unsaturated C_16_ and C_18_ acyl-ACPs are made and incorporated into phospholipids. The compounds 2-methylketone, 3-keto fatty acid, and fatty acid are denoted as MK, KFA, and FA, respectively. The arrows between each intermediate denote, in order, 3-ketoacyl-ACP synthase (FabB or FabF), 3-ketoacyl-ACP reductase (FabG), 3-hydroxyacyl-ACP dehydratase (FabZ), and enoyl-ACP reductase (FabI).

Biochemical evidence of a correlation between acyl-ACP thioesterase specificity and fatty acid profile of the organism from which the enzyme was sourced has been reported previously [14,15]. As an example, the range of 2-methylketones profiled following heating of the culture media of *E. coli* expressing ShMKS2 is very similar to that seen in *S. habrochaites glabratum* trichomes, with 2-tridecanone (13:0 MK) and 2-undecanone (11:0 MK) being the most abundant, suggesting that ShMKS2 shows a preference for similar chain-length intermediates in both plants and *E. coli* [9]. In this study, the substrate preference of GmMKS2-X2 towards unsaturated acyl-ACP substrates might be related to the high percentage of unsaturated fatty acids found in soybean oil. However, GmMKS2-X2 predominantly acts on C14 substrates in *E. coli*, whereas the major unsaturated fatty acids in soybean oils are linoleic acid (18:2 FA), linolenic acid (18:3 FA), and oleic acid (18:1 FA) [13], suggesting that soybean might possess additional acyl-ACP thioesterases that contribute to its fatty acid profile or that *E. coli* might not contain the same range of fatty acids present in soybean. In line with this, substantial amounts of 2-tridecenone (13:1 MK) and some 2-pentadecenone (15:1 MK) are also found in ShMKS2-expressing *E. coli* cultures, whereas these compounds are not detectable in *S. habrochaites glabratum* trichomes [31]. Furthermore, regarding the unsaturated fatty acid biosynthesis pathway, plants possess acyl-ACP desaturases that introduce double bonds into acyl chains in an oxygen-dependent reaction, whereas many bacteria, including *E. coli,* undergo anaerobic desaturation in which the double bond is inserted at the C10 level by 3-hydroxydecanoyl-ACP dehydratase/isomerase (FabA) and is retained in the subsequent C2 elongation cycles [32,33]. Because 3-keto-*cis*-Δ*^3^*-decenoyl-ACP*,* 3-keto-*cis*-Δ*^5^*-dodecenoyl-ACP, 3-keto-*cis*-Δ^7^-tetradecenoyl-ACP, and 3-keto-*cis*-Δ^9^-hexadecenoyl-ACP are intermediates of the unsaturated fatty acid biosynthesis pathway in *E. coli* [33], the unsaturated 3-ketoacids (also called 3-oxo acids) produced by 3-ketoacyl-ACP thioesterases are probably derived from those intermediates and thus have a double bond in the *cis* conformation at the ω-7 position (Figure 6). Although the double bond (C=C) position within the products was not conclusively determined by GC–MS in this study, double bonds in unsaturated fatty acids generated in *E. coli* expressing an acyl-ACP thioesterase from camphor (*Cinnamonum camphorum*) were all in *cis* conformation and at the seventh carbon atom from the methyl end (–CH_3_) of the carbon chain [34]. In agreement, *E. coli* strains overexpressing a native thioesterase (FadM) produced some monosaturated methylketones, such as 13:1 and 15:1, and the latter has been identified as (*Z*)-8-pentadecen-2-one with a double bond (C=C) at the ꞷ-7 position [16]. As unsaturation enhances the fluidity of fatty acids and their derivatives [35], the capacity of GmMKS2-X2 protein to act preferentially on a specific unsaturated substrate in *E. coli* and thus yield unsaturated methylketone 13:1 as a primary product makes it particularly important for engineering the fatty acid biosynthesis pathway towards the production of methylketone-based biofuels.

Heterologous expression of GmMKS2-X2 in *E. coli* also led to the production of *cis*-Δ5-dodecenoic acid and several 3-hydroxyacids such as β-hydroxyoctanoic acid (β-OH 8:0 FA), 3-hydroxydecanoic acid (β-OH 10:0 FA), and β-hydroxydodecanoic acid (β-OH 12:0 FA), in addition to the 3-ketoacids (Figure 3). Notably, there was no direct relationship between β-ketoacid profiles and β-hydroxyacid profiles in the culture media of *E. coli* cells expressing GmMKS2-X2. The 14:1 β-ketoacid is predominantly found in the former, but its β-hydroxyacid form, 14:1 β-hydroxyacid, is negligible from the latter, suggesting that the β-hydroxyacids detected were not directly derived from reducing the corresponding β-ketoacids during the sample handling process, but were likely the products generated by enzymatic catalysis. This points out the possibility that GmMKS2-X2 is also capable of hydrolyzing *cis*-Δ5-dodecenoyl-ACP and 3-hydroxyacyl-ACPs, other types of intermediates formed during fatty acid biosynthesis, to form the corresponding fatty acids, at least when expressed in *E. coli* (Figure 6).

### 3.3. Possible Biological Roles of GmMKS2 in Soybean

In this study, three *GmMKS2* transcripts from *G. max*, *GmMKS2-X1, GmMKS2-X2*, and *GmMKS2-X3*, were identified. The main transcript of *GmMKS2*, *GmMKS2-X2*, is conserved in many plants and widely expressed in multiple soybean tissues. The protein encoded by the main transcript *GmMKS2-X2* is most closely related to *Arabidopsis* ALT3 with 63.2% of sequence identity between the two proteins [10]. GmMKS2-X2 showed thioesterase activity towards a wide range of acyl-ACPs. The alternatively spliced isoform GmMKS2-X1 failed to generate free fatty acids when expressed in *E. coli,* despite the fact that it possesses the conserved Asp residue and the complete C-terminal region important for thioester bond cleavage. While this study focused on 3-ketoacyl-ACP/ 3-hydroxyacyl-ACP thioesterase activity, it remains possible that GmMKS2-X1 may act on other types of acyl-ACPs that are not endogenously abundant in the *E. coli* strain C41(DE3) used. In addition, the observation that this transcript variant is highly expressed in the soybean root raises the possibility that it still may be functionally important. GmMKS2-X3 has a truncation of 71 amino acids encoded by exons 4 and 5 that is replaced by a new stretch of 30 amino acids encoded by the third intron. The typical hotdog fold domain of ALT/MKS2-like thioesterases is encoded by exons 2, 3, 4 and by the first 36 nucleotides of exon 5 (Supplementary File 5). In eggplant (*Solanum melongena*), SmMKS2–3 lost 37 residues in the C-terminal region of the hotdog domain because of the presence of a premature termination codon and it is non-functional [29]. Therefore, it is not surprising that we observed complete loss of thioesterase activity in GmMKS2-X3 when the hotdog-fold domain of this protein was also truncated at the C terminal end. Switching the C terminus from GmMKS2-X2 to GmMKS2-X3 and vice versa to see if there is any change in the activity of the engineered protein compared to its original version may give more elucidative conclusions.

In the wild tomato species (*S. habrochaites*) that contains high levels of 2-methylketones in their trichomes, methylketone synthase 2 (ShMKS2) hydrolyzes 3-ketoacyl-ACP to generate 3-ketoacids, and methylketone synthase 1 (ShMKS1) catalyzes the decarboxylation of those 3-ketoacids to form 2-methylketone products. The 2-methylketones, primarily 2-undecanone and 2-tridecanone, from wild tomatoes act as natural insecticides [11]. Although the biological function of ShMKS2 homologs in other plant species remains to be elucidated, there is cumulative evidence that suggests a defensive role for MKS2/ALT-like proteins in those plants [10,17,19,29]. In eggplants, artificial wounding, methyl jasmonate (MeJA) treatment, or methyl salicylate (MeSA) treatment upregulated the transcriptional expression of *SmMKS2-1* gene in the leaves [29]. In *Arabidopsis,* herbivore-damaged plants emitted more 2-pentanone than undamaged plants [36]. In addition to the protective role against herbivorous insects, 2-tridecanone can also affect surface mobility and biofilm formation of both pathogenic and symbiotic bacteria and as a result, interfere with the bacterial colonization of plant roots [19]. In this study, we also observed that the main transcript of *GmMKS2*, *GmMKS2-X2*, was universally present in all tissues examined, but its expression was the highest in the roots. Furthermore, *GmMKS2-X1*, one of other two alternatively spliced transcripts of *GmMKS2*, was mostly detected in the roots (Figure 4). This raises the questions of whether *GmMKS2* is involved in 2-methylketone biosynthesis in soybean roots, albeit at very low levels, and of the possible interference exerted by 2-methylketone in the plant–symbiont–pathogen interactions.

To further examine the possible roles of the *GmMKS2* gene in the plant, methylketone and fatty acid profiles were analyzed via GC–MS. The predominant FAs present in soybean roots are palmitic acid (16:0), linoleic acid (18:2), linolenic acid (18:3), oleic acid (18:1), and stearic acid (18:0) (Supplementary File 8). Other FAs, such as 8:0, 10:0, 12:0, 14:0, 20:0, and 20:1 are present in smaller quantities. However, we failed to detect the presence of 2-methylketones and 3-hydroxyacids in the roots. An assessment of methylketone and/or 3-hydroxyacid production in wildtype and mutants overexpressing GmMKS2-X2 would be informative to better understand the roles of this enzyme in the crop. Nevertheless, we cannot rule out the possibility that 3-ketoacids and 3-hydroxyacids are produced in the plant and then further metabolized to the other products, whose detection requires additional experiments.

The control of gene transcription via *cis*-regulatory elements in promoters is one of the main modes of gene expression regulation. Induced responses to stresses are often attributed to genes that contain characteristic *cis*-acting elements in their promoters. In silico analysis of the *GmMKS2* promoter sequence revealed a substantial enrichment of *cis*-acting elements related to disease-resistance responses such as ElRE, GT-1 motif, WB-box, and WB-box (Figure 5 and Supplementary File 6). The W-box [(T)TGAC(C/T)] and WB-box [TTTGAC(T/C)] are frequently found in the promoters of various genes that respond to wounding and/or pathogens [37]. The WRKY transcription factors possess one or two highly conserved WRKY domains that can bind to the W-box in the promoter of the target genes to regulate transcription. Many WRKY family proteins were shown to be involved particularly in plant defense against attack from pathogens [38,39,40,41]. Furthermore, inspection of the 5′ upstream region of *GmMKS2* also revealed the presence of multiple *cis*-regulatory elements related to abiotic stress signaling pathways, such as E-box, G-box, ACGT element, ABRE (ABA-responsive element)-like sequence, SRE (sugar-responsive element), I-box, DRE (dehydration-responsive element) core motif, CCAAT box, and MYB-related motifs (CNGTTR, YAACKG). Many of those motifs appeared more than once in the 1 kb promoter of the gene (Supplementary File 6). The E-box, with the G-box (CACGTG) being the most common variant, is specifically bound by transcription factors of the bZIP and bHLH families (Figure 5) [42,43]. In plants, bZIP and bHLH proteins are important regulators of plant growth, metabolism, and responses to abiotic and biotic stimuli [44,45,46,47,48]. Many of the identified bHLH proteins bind to the E-box and regulate abiotic stress-responsive genes, such as *RD* (responsive to dehydration) and *ERD* (early responsive to dehydration) [49,50], and genes involved in the biosynthesis of secondary metabolites [51,52,53]. WRKY proteins, which specifically recognize the W-box, also play an important role in the abiotic stress responses of soybean plants [54,55]. A high percentage (~50%) of *cis*-regulatory elements identified in the *GmMKS2* promoter are abiotic stress-responsive motifs, suggesting that *GmMKS2* might be regulated by certain abiotic stressors as well. In fact, the regulation of lipid-modifying enzymes at the transcriptional level is employed by plants to adapt to stress conditions [56]. It is clear that a variation in membrane lipid composition can alter membrane fluidity and affect a number of plant responses to abiotic stress, especially temperature and osmotic sensitivity [57,58].

A common consequence of most abiotic and biotic stresses is that they result, at some stage of stress exposure, in an excessive accumulation of reactive oxygen species (ROS), leading to a condition of oxidative stress. In line with this, in silico analysis of a co-functional network database for *G. max* at the SoyNet by both “Find new members of a pathway” and “Find context-associated genes” searches [59], using *GmMKS2* (Glyma.01G080400) as a guide gene, identified a new candidate gene (Glyma.10G000900) that is predicted to encode a subunit of the NAD(P)H dehydrogenase (NDH) complex and is annotated as “response to oxidative stress” according to Gene Ontology biological process terms. Taken together, these observations support the possible role of *GmMKS2* gene in plant stress responses. It remains unclear whether alternative splicing of this gene has a stress-related physiological function or not, but it has been reported that stress-related genes from plants are particularly prone to alternative splicing events, which often modulate the ratio between active and non-active isoforms in response to biotic and abiotic stress cues, thus modulating the expression of key regulators in stress signaling [60].

## 4. Materials and Methods

### 4.1. Plant Material and Growth Conditions

Soybean seeds (*G. max* L) were obtained from the Research Institute for Oil and Oil Plants (IOOP) (Vietnam). The seedlings were grown in potting soil under controlled conditions.

### 4.2. Bioinformatics

ShMKS2 sequence was used as a query sequence for TBLASTN searches of William 82 Assembly 2 (Wm82.a2) Genomic Sequence Database (https://soybase.org) to identify an MKS2 homolog gene in soybean. The gene structure was manually curated on the basis of alignments of EST and homologous cDNA sequences to genomic sequence. Multiple sequence alignments were generated using the MUSCLE program at the European Bioinformatics Institute (https://www.ebi.ac.uk/Tools/msa/muscle/) [61], and a rooted phylogenetic tree based on the multiple sequence alignment was built using Molecular Evolutionary Genetics Analysis X (MEGA X) [62].

### 4.3. Identification and Isolation of GmMKS2 Transcripts

RNA was extracted from soybean immature seeds using Ribospin^TM^ Plant Kit (GeneAll Biotechnology Co., Ltd., Seoul, Korea), treated with DNase I to remove genomic DNA contamination, and used as a template in a reverse transcription (RT) reaction to generate cDNA. The presence of two alternatively spliced variants, *GmMKS2-X1* and *GmMKS2-X3*, was identified using a forward primer located in the first exon and a reverse primer located at the 3′-end of intron 3. The full-length sequences of the primary transcript *GmMKS2-X2* and the two alternatively spliced variants *GmMKS2-X1* and *GmMKS2-X3* were amplified from the cDNA using the same pair of primers located in the 5′ untranslated region (UTR) and 3′ UTR (Supplementary File 2). PCR amplification was performed with the Phusion High-Fidelity DNA Polymerase (Thermo Scientific, Massachusetts, USA) and the following PCR conditions: 98 °C for 30 s, followed by 35 cycles at 98 °C for 10 s, 55 °C for 20 s, and 72 °C for 30 s, and a final extension step at 72 °C for 5 min. The expected PCR products were cloned into the pJET1.2/blunt cloning vector (Thermo Scientific, Massachusetts, USA), and their sequences were verified.

### 4.4. Expression of GmMKS2 Isoforms in E. coli

The coding sequence of each GmMKS2 isoform (without the transit peptide-encoding region) was amplified by PCR and inserted between the *Nde*I and *Xho*I sites of the bacterial expression vector pETDuet-1 (Invitrogen, California, USA) to generate the recombinant plasmid pETDuet-1-GmMKS2-Xn (n = 1, 2 or 3). Primers for cloning the region encoding the mature GmMKS2-Xn into pETDuet-1 are shown in Supplementary File 2. The recombinant plasmid was introduced into *E. coli* C41(DE3) cells (Lucigen, Wisconsin, USA). The *E. coli* C41(DE3) cells containing the plasmid pETDuet-1-GmMKS2-Xn were grown in Terrific Broth (TB) medium containing 100 ng/mL ampicillin, and gene expression was induced by the addition of 0.5 mM isopropylthio-β-galactoside (IPTG) after the culture optical density at 600 nm had reached 0.6 to 0.8. After induction with IPTG and growth at 18 °C for 16 h, the culture was collected for further analysis. Negative control experiments (induced cells with either the empty plasmid pETDuet-1 or the pETDuet-1-GmMKS2-X2-D81A plasmid) were performed in parallel. The cell cultures were centrifuged at 8000 rpm and the pellets were resuspended in lysis buffer (100 mM NaCl, 10 mM Tris, 1 mM EDTA, 10% glycerol, pH 8). Samples of total cell lysate and soluble and insoluble fractions were separated on a 12% SDS-PAGE gel under reducing conditions and stained with Coomassie Blue.

### 4.5. Profiling 3-Ketoacids in Bacterial Culture Media and in Soybean Roots via Identification and Quantification of Their Decarboxylated Products

GmMKS2 protein expression was induced in bacterial cultures as described above. As 3-ketoacids are too unstable to be detected by GC–MS or GC–FID, they were decarboxylated to 2-methylketones by heating at 75 °C for 30 min followed by cooling at 30 °C for 30 min. The resulting 2-methylketones were then extracted from the heated bacterial culture media with hexane. For 2-methylketone profiling of soybean (*G. max*) roots, approximately 500 mg of roots of intact six-week-old soybean plants was frozen, ground, and submerged in 2 mL of hexane for 5 h at 45 °C. The compound 2-dodecanone was added as an internal standard before extraction. Then, 1 μL of the hexane extract was injected into an HP5 MS column (Agilent Technologies; 30 m length, 0.25 μm film thickness, and 0.25 mm I.D.) of a GC Agilent 6890N (Agilent Technologies, California, USA) coupled to an Agilent 5972 Mass Spectrum Detector. The carrier gas (helium) was used at a constant flow rate of 1 mL/min. The injector was programmed to hold at 250 °C for 30 s, ramp to 280 °C at 50 °C/min, and hold for 1 min. The column oven was initially at 60 °C for 5 min, then was increased at 15 °C/min up to 300 °C. The identification of saturated methylketones was done by comparing retention times and MS spectra of the peaks in each sample to those of authentic standards: 2 heptanone (7C), 2-nonanone (9C), 2-undecanone (11C), 2-tridecanone (13C), and 2-heptadecanone (17C) (Sigma-Aldrich, Missouri, USA). The determination of unsaturated methylketones and 2-pentadecanone was based upon their spectra which have been previously described in detail [10,16].

### 4.6. Determination of Free Fatty Acids in Bacterial Culture Media and in Soybean Roots

The free fatty acids secreted into the culture media of *E. coli* carrying an empty vector or expressing GmMKS2-X1, GmMKS2-X2, or GmMKS2-X3 were methyl esterified and analyzed via by GC–MS. Esterification of free fatty acids in bacterial culture media was performed as described previously [12], with minor modifications as follows. Cells from 15 mL of each bacterial culture were pelleted via centrifugation at 4500× *g*. Then, 1 mL of culture supernatant was mixed with 2 mL of chloroform/methanol (1:1, *v*/*v*) and 44 μL glacial acetic acid. Lipids were extracted by vortexing for 10 s, and phases were separated by centrifugation at 4500× *g*. The lower phase was transferred to a clean glass tube, and excess solvent was removed by a rotary evaporator. Samples were resuspended in 5 mL of 1N methanol/HCl anhydrous solution and incubated at 60 °C for 2 h [63]. The reaction was cooled to room temperature, and 5 mL of 0.9% NaCI was added to stop the esterification reaction. Fatty acid methyl esters were then extracted into 1 mL hexane by vortexing for 30 s, and phases were separated by centrifugation. Similarly, free fatty acids were extracted from 500 mg of roots of intact six-week-old soybean plants and methyl-esterified as described above. The hexane extract was then used for GC–MS analysis.

A SCION 456 gas chromatography system equipped with an RXI-5MS column (30 m length, 0.25 mm inner diameter, 0.25 μm film thickness) and a Scion mass spectrometer (70 eV, mass-to-charge ratio 40–500) were used for identification by MS. Samples were injected in splitless mode, with an injector temperature of 230 °C and a solvent delay of 4 min. The carrier gas was helium at a constant flow rate of 2 mL/min. The column oven temperature was initially held at 50 °C, then increased at a rate of 30 °C/min up to 280 °C, which was held for 5 min. All fatty acid methyl esters were identified via their mass spectra in comparison to standard spectra from the National Institute of Standards and Technology (NIST) Mass Spectral Library (version 2.2) and/or their retention times in comparison to those of authentic standards.

### 4.7. In Silico Analysis of Regulatory Elements in the Promoter Region of GmMKS2 Gene

The transcription start site was predicted by the TSSPlant program available at the Softberry platform (http://www.softberry.com) [23]. For in silico analysis of the promoter sequence, we amplified the [−1000: +101] region of the *GmMKS2* gene, where +1 corresponds to a transcription start site of the gene, using the primer reported in Supplementary File 2. The promoter region was sequenced and analyzed for potential regulatory motifs using the software programs NSITE-PL (http://www.softberry.com) and PLACE database [25].

### 4.8. Gene Expression Analysis by qRT- PCR

Different tissues of *G. max* plants were collected and immediately stored in liquid nitrogen. Total RNA was isolated using EZ-10 Spin column Plant RNA Mini-preps Kit (Bio Basic Inc., Ontario, Canada) and treated with RNase-free DNase I to remove contaminating DNA using RapidOut DNA Removal Kit (Thermo Scientific, Massachusetts, USA). RNA concentration was determined by a UV–Vis spectrophotometer. cDNA was synthesized from 1 µg of DNase-treated total RNA in a 20 µl reaction using RevertAid First Strand cDNA Synthesis Kit (Thermo Scientific, Massachusetts, USA). To quantify the mRNA abundance of *GmMKS2*, quantitative RT-PCR was performed as previously described [64]. To this aim, 1 μL of cDNA was used as a template for PCR amplification in a 20 μL reaction using SolGent^TM^ h-Taq DNA Polymerase (SolGent, Daejeon, Korea), EvaGreen^TM^ Dye (Biotium, California, USA), and gene-specific primers (Supplementary File 2). The reactions were performed in a Lightcycler 96 system (Roche, Basel, Switzerland) with the following cycles: 95 °C for 20 s, followed by 40 cycles (95 °C for 30 s, 58 °C for 40 s, and 72 °C for 10 s). A final dissociation step was performed to assess the quality of the amplification product. Relative expression levels of each of the three *GmMKS2* variants in different tissues were calculated using the relative quantification method normalized to the expression levels of soybean F-box protein SKIP-like (*cons6*; GenBank: NM_001254106). Each data point represents an average of three biological replicates.

### 4.9. Accession Number

*GmMKS2* gene sequence can be found in Genbank under the accession number MN364665.

## 5. Conclusions

The soybean (*G. max*) contains only a single functional ortholog, designated GmMKS2, of the wild tomato methylketone synthase 2 (ShMKS2). The main transcript of *GmMKS2*, *GmMKS2-X2,* is widely expressed in plant tissues, and the protein encoded by this transcript showed a broad catalytic activity, producing a wide range of fatty acids varying considerably in terms of chain length, oxidation state, and saturation level, with 14:1 3-ketoacid being the main product. *GmMKS2* underwent alternative splicing to produce two additional alternatively spliced transcripts, *GmMKS2-X1* and *GmMKS2-X3*, but neither encodes a protein with thioesterase activity when recombinantly expressed in *E. coli.* The results of in silico analysis of the *GmMKS2* promoter sequence for the presence of putative *cis*-regulatory elements and of the analysis of a publicly available co-functional network database for the gene co-regulated with *GmMKS2* support a stress-related physiological function for this gene. However, it would be necessary to perform RT-PCR analysis to experimentally verify these predictions and study the genotype–phenotype correlation in order to better understand the detailed roles of this gene in the development of soybeans, particularly under abiotic and biotic stresses.

## Figures and Tables

**Table 1:** Quantification of 2-methylketones detected following heating of the culture media of *E. coli* cells expressing the main isoform of GmMKS2 (GmMKS2-X2). Cells were grown, and media were collected and treated as described in “Materials and Methods.” The products 2-methylketones, which were derived from 3-ketoacids, were quantified by comparison of the peak areas with those of a linalool internal standard using GC. Values are averages ± SE calculated from three replicates (R1, R2, and R3). Significant differences were assessed by one-way ANOVA followed by Tukey’s HSD (honest significant difference) test. Means with different letters are significantly different (*p* < 0.005).

Retention Time (min)	3-Ketoacids Secreted into Media of *E. coli* Cells Expressing GmMKS2-X2	2-MKs Detected by GC	2-Methylketone Accumulation (Ng/OD Unit)			
			R1	R2	R3	Mean ± SE
4.340	3-oxooctanoic acid	7:0 MK	619.02	547.27	576.02	580.77 ± 20.85 ^b^
6.707	3-oxodecanoic acid	9:0 MK	210.98	203.95	195.00	203.31 ± 4.62 ^c^
9.905	3-oxododecenoic acid	11:1 MK	206.85	227.07	104.41	179.44 ± 37.97 ^c^
10.039	3-oxododecanoic acid	11:0 MK	521.58	529.29	467.29	506.06 ± 19.51 ^b^
12.159	3-oxotetradecenoic acid	13:1 MK	5815.96	6091.41	6161.55	6022.97 ± 105.14 ^a^
12.390	3-oxotetradecanoic acid	13:0 MK	199.25	222.36	173.19	198.27 ± 14.20 ^c^
13.867	3-oxohexadecenoic acid	15:1 MK	141.92	151.64	118.91	137.49 ± 9.71 ^c^
13.786	3-oxohexadecanoic acid	15:0 MK	98.18	72.29	34.47	68.31 ± 18.50 ^c^
15.066	3-oxooctadecenoic acid	17:1 MK	172.27	172.08	151.65	165.33 ± 6.96 ^c^

## Supplementary Materials

The following are available online at https://www.mdpi.com/2223-7747/8/10/397/s1. Supplementary File 1: Nucleotide sequence of *GmMKS2* obtained by PCR and verified by sequencing. Shown here are 1105 nucleotides upstream of the translation start codon (ATG), five exons, and four introns. The boxed nucleotide located at −106 bp upstream of the initiating codon ATG was predicted to be the transcription start site (A_+1_). The putative promoter region spanning from −1000 to +101 relative to the transcription start site is highlighted in grey. Start and stop codons are in bold. Exons are underlined. Supplementary File 2: Synthetic oligonucleotide used in this study. Start codons and stop codons are in bold. Restriction sites on primers are underlined. A brief description of what each primer was used for is provided. Supplementary File 3: Alignment of GmMKS2-X1, GmMKS2-X2, and GmMKS2-X3 transcript sequences and of protein sequences encoded by these transcripts. (a) Alignment of three *GmMKS2* transcript variants. The region of intron 3 inserted between exon 3 and exon 4 to generate two novel splice variants *GmMKS2-X1* and *GmMKS2-X3* are boxed. Stop codons are in bold; (b) Alignment of three GmMKS2 isoforms. Identical residues are shaded in black, and similar residues are shaded in grey. Supplementary File 4: SDS-PAGE analysis of soluble (S) and insoluble (I) fractions of *E. coli* cells carrying an empty plasmid (pETDuet-1), or expressing GmMKS2-X1, GmMKS2-X2, GmMKS2-X3, or GmMKS2-X2-D81A. Supplementary File 5: Amino acid sequence alignment of GmMKS2 isoforms with functionally characterized MKS2s from *Solanum habrochaites*, *Solanum lycopersicum*, *Arabidopsis thaliana*, and *Pseudomonas* species (Ps). Accession numbers are as follows: GmMKS2, MN364665; AtALT1, At1g35290; AtALT2, At1g35250; AtALT3, At1g68260; AtALT4, At1g68280; ShMKS2, GU987106; SlMKS2a, GU987112; SlMKS2b, GU9877113; SlMKS2c, GU987114; Ps4HBT, EF569604. Identical residues are shaded in black, and similar residues are shaded in grey. The black horizontal line indicates the conserved hotdog-fold region present in MKS2s/ALTs. The conserved Asp residue is indicated by a star symbol. Supplementary File 6: Putative *cis-*regulatory elements in the GmMKS2 promoter identified through PLACE database [65,66,67,68,69,70,71,72,73,74,75,76,77,78,79,80,81,82,83,84,85,86,87,88,89,90,91,92,93,94,95]. Supplementary File 7: Phylogenetic tree of the soybean GmMKS2 proteins and previously characterized MKS2s from *S. habrochaites* (ShMKS2), *S. lycopersicum* (SlMKS2a, SlMKS2b, and SlMKS2c), *A. thaliana* (AtALT1-4), and *Pseudomonas* species (Ps4HBT). Bootstrap values were determined with 1000 replicates and are indicated next to the branches. Evolutionary analyses were conducted in MEGA X [62]. Supplementary File 8: Analysis by gas chromatography–mass spectrometry of 2-methylketones (a) and methyl esters of fatty acids (b) from soybean roots. Peaks were identified by mass spectrometry (MS), and their retention times were compared to those of known compounds. Extracted ion chromatogram (EIC) at *m*/*z* 58 for the detection of 2-methylketones shows a single peak corresponding to the internal standard 2-dodecanone.

## Abbreviations

ACP	acyl carrier protein
ALT	acyl-lipid thioesterase
ANOVA	analysis of variance
APRT	adenine phosphoribosyl transferase
BLAST	basic local alignment search tool
FAS	fatty acid synthase
HSD	honest significant difference
IPTG	isopropylthio-β-galactoside
MeJA	methyl jasmonate
MeSA	methyl salicylate
MKS2	methylketone synthase 2
MKS1	methylketone synthase 1
ORF	open reading frame
PAGE	polyacrylamide gel electrophoresis
qRT-PCR	quantitative real-time polymerase chain reaction
SDS	sodium dodecyl sulfate
SE	standard error
TE	thioesterase
TSA	transcriptome shortgun assembly
