# Supplementary material for: Identification and Functional Characterization of a Soybean (Glycine max) Thioesterase that Acts on Intermediates of Fatty Acid Biosynthesis
Source: Plants (Basel). 2019 Oct 8;8(10):397. doi: 10.3390/plants8100397 (PMC6843456; doi:10.3390/plants8100397)
Supplement: Supplementary file 1 [file plants-08-00397-s001.zip › Supplementary Files_proofed/Supplementary File 1-revised.pdf]

Supplementary File 1. Nucleotide sequence of *GmMKS2* obtained by PCR and verified by sequencing. Shown here are 1105 nucleotides upstream of the translation start codon (ATG), five exons, and four introns. The boxed nucleotide located at −106 bp upstream of the initiating codon ATG was predicted to be the transcription start site (A_{+1}). The putative promoter region spanning from −1000 to +101 relative to the transcription start site is highlighted in grey. Start and stop codons are in bold. Exons are underlined.

GAAGTGTAGCATCTTTCAACGTTTTAGGAAACACTTAACACATGATTATGTCATCATTCTTAA

ACAAATTCATCTGTGTAACAAATGCTTCCACGTGTTCATCTGGGTCCAAGAGTCCATCGTAAA

GATCTATGGTCAACATCTTCCATTCGGGCGGGAGTTGTGCTTCCATAATACCATCAATAAAGG

GATAAAGTCGATTTTTGGCATTTGTTGCTCTTGGGGTTGGTTGTGTGATAGAGGGAGTTCTTTC

TCTCCATGTCGTGTTAGCCTCCTCAGTCCTGGTTGCATTATGGTCTTCGTGAAGCTTCAGGTAC

TGGTCCTTAAGGGTGGCATTTTCCTTTAGAGGTTCTCCATTTCTTGTGCGTTGTTTCACTTGAGC

ATGTTCATCTCTTTTTGCATGGCCACTATCATAGCATGATCATCGAGCTCCTGATGCAGGATAT

TCTCCCCATTCATTGGGTTCCCTTCTTTAGCGACTCCCATGACAGGCATTTTCAAGCATGGTGA

ATGCTTGTCCCAGTTGTTGATTTCCATGATAGGCAACATTGGTGTTGTCACCATTCACAACCAT

TGATAATGTAGTGGTCAAAGGAGAAGATTGCCTTCAGACCCCACAGTAGGCACCAAATATTC

TTACCGGATTTGGCAGAACTTGCTCACAATGATAATATATGTTTTTAGAGGTTACATCTATCTT

TCTTATAGAAGATAGAGCTTTCATATTACCTTAATTATACTCTAGATCCGAGTAGCCTGTTCGA

TTGGCTAGTGAATACATAGGCTTGTTGAGTCGAAATAGTACAATTTATTATAAATTTTTGTTAT

ATAATTCTATATTAGTTCTTTTTAAATGATTATAAAAAGAAATTATTCAAGAGTGATATCTATT

ACCAACATTTCGTTGATGTGAGATTGTTTTAGAAAATTTGAAATTTGAAGTTTTATGAATGAA

AAAAAAAGTGATAACTAAAAAAAAAGCAAAACAAATTAGGTATTCAAAAATTTTGCTAGGCA

AAGGTCGTTGAGGTTGAGGAAAAAAAAAGTATAATAGCAGTAGCATGGGTTGTAACGCCTATA

ATCATAATCCCGTTGTAGTTCCAAAAA**ATG**AGCTACAACCACACTTCCTCGATGTCATTGCCT

TCCCCATTGTACCTGAATACTACGTCGTTTCGCCTCACGCGCCAATCTCCTTTTCCTTTTCCCCG

CCGGCGCTTCAATCCACCGGCTTTCCGATCAGTTTCGCCGTTGAGTTCCAGCCCCTCTGCATCA

CTCTTCGATCTCAGAGGGGGCAAAGGGTAATTTCCTCACACAACCCTTGTTTGTGCAATCGAT

TCCGTTAATTTTGTTGCCTTTTCATGTGATTCGAATCGAGCATCTCTTTTTTAAATTCGTGATTA

ATTTATTCTATATATAATAATATTCTTCTTACAATTTATAACAGAATGAGTGGATTCCATGACG
TTGAACTGAAGGTGCGCGACTATGAGTTGGATCAGTACGGTGTGGTTAACAATGCAGTTTATG
CTAGTTATTGCCAGCACGGTGAGACCTCTTGAGCTGGTTCTTACTGTTGCTTGCTTGCTTAAAT
CAATGTTTCCCTTTTACTGGTAATGTCGTATGTGTATATATTTCTCTTGTAATAGATGTTAATTT
TGAAATGTTTATAACTCCAAATGAGCACTAAGTTTTGACATGGAATAATTGTCGACAATAGTT
ATGCCATGAGTTAGCAATTTTAACAACAGTTATTGTCTGCTTTTCTAGGTATATCAAAATTGTA
ATCTTTGATATCTTAGTCCTTTTGTTTATGCTACAAAATTATATTTTGATTTATGTTCTTTAAAAC
TTCAAATTGAATTTAGATTCGACATATTTTTAGAGTATAAAAAAATTATGAAGGTAAAACAAC
ATAATCAGACTAGCACAGACATATGATAAAAGTTTTATAATCTTTCTTCTCTTTTCCTTATAAA
ACAAATGTATAGGCTGTGATAGTAATATTATGAAGGGATTTTATGGGAAAACATTCTGAACA
TAGTTGGAAATGAAACAAAAGAGCAAGAAGTAAAGGAATCAGATCATAATTTTTAAACAAA
TACTGCTGTTTCCCGGTAAAACCATTAGAAGGCTCACATGTAGTTTTATTGAAGCATATTCCCC
AGATTAGCATCTTTCTGAAAGAACAATTCTCAATGTTAGTATGCAAGATAAAGTACAGTATAC
TTCATATGCAGAAAGCTGTCACACTTTGTGAGGAATGAACTTTCATACATACATATGAACATT
TGTTAGTCTATATGAAAATATAAAATAGGCAATTGAGAAGGAGAAAGAACAATACAGAAAA
TATAGTGTTAGGATCCTGAAAAAATAATTAATCAGAATGGATAAAATGGACAAACAACAGA
GAATCTGGCATCTGTGTTTAGTATTACTAACTCCAGAATAGTGATTCTGGCTTCTGAGTATTTC
TTGGAATGCAAGAATGACAGAACGAGAAAAAAAACAGAAAAGAAATGACTCATTCCAAGC
ATTGGTTTGGAACTTCCCTATCCAAGCATTTAATTCATCAATTGGGAAGAAATACCCTGTGGA
GCCTGACTCAACGAGTATTGCCAATGCAAGGCTATTTGTAGTATGGAACTGAGTGATGTCTAC
ACGAATAATCCTTTAGTCATACCAATATTCCACCGCCTTCTTCACCCTTTACTCGTTAAAATAA
AACTACATTAGGATTTTGCAGGTTCACATCAAAACTTTTCTATTCAAATTTAGATGAAATCTA
ATGGTTCATCTCACTGATAAGTTCTATTTGTTTCTCTAAGAGTTGTGTATAGTATTTTTTCTTATC
TTGTATATGCTTGGAAATATTTCCCCCCCTTTTCTTGATCTCTGTTTTGGTATATTTGAGCCAGT
ATAATTGTTATGATCAGGTGGGAAGTTGGTGAGTTCTTATGGTACTTTGGTATTTGTTTTGGTTG
GGGTCACTTTTCTAATTTATTCCGTTGGACCTGGGATTCGAGTACATGGGAGGTGATTACAAT

GGGTCAATGATGACTATAGTTACTGTGAGGGCAGTTTGGTGGGAGGTATTGTAACCTGGGTTA
TTTCCTATTTATACTACATTGTTAATGGGGCATAACCTAGCATGGCAGGGTACTGTGCTTTATT
ACTATGTCTGTATTTTTTGGTGCTTCTGGTACCAAGTATTAATATATTAATTATCTTTTGCTGAT
AAAAAAAGTTATGATCAGGGTATTGACACTTGACAGGATACTGAATTGAAACGTATTGAGTT
GAATGGTCTGCAAACTTTTTAAGTTATCCTTTGTACATTTGTGGCAATTAAAAGGCATGCTTTC
AATTTTAGATGTATAAACTGATCTATTGCCATATATATTTTCATAAGGTCGTCATGAACTCTTG
CAAAACATTGGTATTAATTGCGATGCTGTGGCTCGCAGTGGTGATGCATTGGCATTGTCTGAA
CTATCGCTCAAATTCCTTGCACCTCTAAGAGTAAGCCTCTCCAATTTTGCTTGTCATTTCTGTTT
GTTAGGATATAGTGCAACTGTGCAAATAACCTCCTTATACATTTTAGTACACTAAAAGATATG
TTTTTCTGTATAAGTTAGTTCTGTTTTCCAATATTTGTTCTCATAATAAGAACATGCAACCAGA
GTGGAGACAAATTTGTTGTAAGAGTTAGGATTTCTGGCTCTTCAGCTGCTCGTTTATACTTTGA
TCACTTCATCTATAAGCTGCCAAACCAAGAGGTCTTTCTCCACCACTTGCTGCTTATTGTATTT
CCATTTTTGAGAATATTTGCTGATATTTGGTGAAATGGTGGCAGCCTATTTTGGAAGCCAAGG
CCATAGCGGTGTGGCTTGACAAAAACTATCGTCCTATACGAATTCCAGCAGAGATGAAGTCT
AAATTTGTAAAGTTTATTCGAATTGAGGACTCT**TAA**TTCAGTTACCAGTTGAGTATAGCTTATT
GGTTAATAGAGCATTAAGAAAGGTAATTATTTATGTA
