# Supplementary material for: Identification and Functional Characterization of a Soybean (Glycine max) Thioesterase that Acts on Intermediates of Fatty Acid Biosynthesis
Source: Plants (Basel). 2019 Oct 8;8(10):397. doi: 10.3390/plants8100397 (PMC6843456; doi:10.3390/plants8100397)
Supplement: Supplementary file 1 [file plants-08-00397-s001.zip › Supplementary Files_proofed/Supplementary File 2_revised.pdf]

Supplementary File 2. Synthetic oligonucleotide used in this study. Start codons and stop codons are in bold. Restriction sites on primers are underlined. A brief description of what each primer was used for is provided.

Primer name	Primer sequence (5′-3′)	Brief description
**Isolation of *GmMKS2* promoter region**		
Promoter-GmMKS2-F	CTTGATGTTGGCGAATGC	Forward primer located in the 5′UTR of *GmMKS2* gene
Promoter-GmMKS2-R	CAACGGCGAAACTGATCG	Reverse primer located in the first exon of *GmMKS2* gene
**Detection of *GmMKS2* alternative splicing variants (*GmMKS2-X1* and *GmMKS2-X3*)**		
GmMKS2-Met-F	CCATGG**ATG**AGCTACAACCACACTTC	Forward primer located in the first exon of *GmMKS2* gene
GmMKS2-45nt-R	CTGGTTGCATGTTCTTATTATG	Reverse primer located in the last 45 nucleotides of intron 3.
**Isolation of *GmMKS2* transcripts**		
GmMKS2-5′UTR-F	AGGCAAAGGTCGTTGAG	Forward primer for amplifying full-length *GmMKS2-X1, GmMKS2-X2,* and *GmMKS2-X3* variants as well as *GmMKS2* genomic sequence
GmMKS2-3′-UTR-R	GTTACCTCCTTCCTCACTCAC	Reverse primer for amplifying full-length *GmMKS2-X1, GmMKS2-X2,* and *GmMKS2-X3* variants as well as *GmMKS2* genomic sequence
**Real-time PCR analysis**		
GmMKS2-X1-qF	GTATTAATTGCGATGCTGTG	Forward primer for *GmMKS2-X1* qPCR

GmMKS2-X1-qR	CAAATTTGTCTCCACTTCTTAG	Reverse primer for *GmMKS2-X1* qPCR
GmMKS2-X2-qF	ATTGGTATTAATTGCGATGCTG	Forward primer for *GmMKS2-X2* qPCR
GmMKS2-X2-qR	GAGAACAAATATTGGAAAACAGAATC	Reverse primer for *GmMKS2-X2* qPCR
GmMKS2-X3-qF	TAGTTATTGCCAGCACGGTCG	Forward primer for *GmMKS2-X3* qPCR
GmMKS2-X3-qR	GACAAGCAAAATTGGAGAGGC	Reverse primer for *GmMKS2-X3* qPCR
cons6-qF	CTTTTACATTTATACCCGGCAGATTG	Forward primer for *cons6* qPCR
cons6-qR	CCCATCCAGAATCAAAATCAGAAT	Reverse primer for *cons6* qPCR
**Recombinant protein expression**		
*Nde*I-GmMKS2-X1/X2/X3-sf-F	CAT**ATG**AGTGGATTCCATGAC	Forward primer for amplifying the region encoding the mature GmMKS2-X1, GmMKS2-X2, and GmMKS2-X3
*Xho*I-GmMKS2-X1/X2-sf-R	CTCGAG**TTA**AGAGTCCTCAATTC	Reverse primer for amplifying the region encoding the mature GmMKS2-X1 and GmMKS2-X2
*Xho*I-GmMKS2-X3-sf-R	CTCGAG**TTA**GTGTACTAAAATGTATAAGGAGGT	Reverse primer for amplifying the region encoding the mature GmMKS2-X3
