# Supplementary material for: Identification and Functional Characterization of a Soybean (Glycine max) Thioesterase that Acts on Intermediates of Fatty Acid Biosynthesis
Source: Plants (Basel). 2019 Oct 8;8(10):397. doi: 10.3390/plants8100397 (PMC6843456; doi:10.3390/plants8100397)
Supplement: Supplementary file 1 [file plants-08-00397-s001.zip › Supplementary Files_proofed/Supplementary File 3_revised.pdf]

Supplementary File 3: Alignment of GmMKS2-X1, GmMKS2-X2, and GmMKS2-X3 transcript sequences and of protein sequences encoded by these transcripts. (a) Alignment of three *GmMKS2* transcripts. The region of intron 3 inserted between exon 3 and exon 4 to generate *GmMKS2-X1* and *GmMKS2-X3* transcripts are boxed. Stop codons are in bold; (b) Alignment of three GmMKS2 isoforms. Identical residues are shaded in black, and similar residues are shaded in grey.

```
(a)
GmMKS2-X2      ATGAGCTACAACCACACTTCCTCGATGTCATTGCCTTCCCCATTGTACCTGAATACTACG 60
GmMKS2-X1      ATGAGCTACAACCACACTTCCTCGATGTCATTGCCTTCCCCATTGTACCTGAATACTACG 60
GmMKS2-X3      ATGAGCTACAACCACACTTCCTCGATGTCATTGCCTTCCCCATTGTACCTGAATACTACG 60
               ************************************************************

GmMKS2-X2      TCGTTTCGCCTCACGCGCCAATCTCCTTTTCCTTTTCCCCGCCGGCGCTTCAATCCACCG 120
GmMKS2-X1      TCGTTTCGCCTCACGCGCCAATCTCCTTTTCCTTTTCCCCGCCGGCGCTTCAATCCACCG 120
GmMKS2-X3      TCGTTTCGCCTCACGCGCCAATCTCCTTTTCCTTTTCCCCGCCGGCGCTTCAATCCACCG 120
               ************************************************************

GmMKS2-X2      GCTTTCCGATCAGTTTCGCCGTTGAGTTCCAGCCCCTCTGCATCACTCTTCGATCTCAGA 180
GmMKS2-X1      GCTTTCCGATCAGTTTCGCCGTTGAGTTCCAGCCCCTCTGCATCACTCTTCGATCTCAGA 180
GmMKS2-X3      GCTTTCCGATCAGTTTCGCCGTTGAGTTCCAGCCCCTCTGCATCACTCTTCGATCTCAGA 180
               ************************************************************

GmMKS2-X2      GGGGGCAAAGGAATGAGTGGATTCCATGACGTTGAACTGAAGGTGCGCGACTATGAGTTG 240
GmMKS2-X1      GGGGGCAAAGGAATGAGTGGATTCCATGACGTTGAACTGAAGGTGCGCGACTATGAGTTG 240
GmMKS2-X3      GGGGGCAAAGGAATGAGTGGATTCCATGACGTTGAACTGAAGGTGCGCGACTATGAGTTG 240
               ************************************************************

GmMKS2-X2      GATCAGTACGGTGTGGTTAACAATGCAGTTTATGCTAGTTATTGCCAGCACGGTCGTCAT 300
GmMKS2-X1      GATCAGTACGGTGTGGTTAACAATGCAGTTTATGCTAGTTATTGCCAGCACGGTCGTCAT 300
GmMKS2-X3      GATCAGTACGGTGTGGTTAACAATGCAGTTTATGCTAGTTATTGCCAGCACGGTCGTCAT 300
               ************************************************************

GmMKS2-X2      GAACTCTTGCAAAACATTGGTATTAATTGCGATGCTGTGGCTCGCAGTGGTGATGCATTG 360
GmMKS2-X1      GAACTCTTGCAAAACATTGGTATTAATTGCGATGCTGTGGCTCGCAGTGGTGATGCATTG 360
GmMKS2-X3      GAACTCTTGCAAAACATTGGTATTAATTGCGATGCTGTGGCTCGCAGTGGTGATGCATTG 360
               ************************************************************

GmMKS2-X2      GCATTGTCTGAACTATCGCTCAAATTCCTTGCACCTCTAAGA------------------ 402
GmMKS2-X1      GCATTGTCTGAACTATCGCTCAAATTCCTTGCACCTCTAAGA------------------ 402
GmMKS2-X3      GCATTGTCTGAACTATCGCTCAAATTCCTTGCACCTCTAAGAGTAAGCCTCTCCAATTTT 420
               ******************************************

GmMKS2-X2      ------------------------------------------------------------ 402
GmMKS2-X1      ------------------------------------------------------------ 402
GmMKS2-X3      GCTTGTCATTTCTGTTTGTTAGGATATAGTGCAACTGTGCAAATAACCTCCTTATACATT 480

GmMKS2-X2      ------------------------------------------------------------ 402
GmMKS2-X1      --------------------------------------TTCTGTTTTCCAATATTTGTTC 424
GmMKS2-X3      TTAGTACACTAAAAGATATGTTTTTCTGTATAAGTTAGTTCTGTTTTCCAATATTTGTTC 540

GmMKS2-X2      -----------------------AGTGGAGACAAATTTGTTGTAAGAGTTAGGATTTCTG 439
GmMKS2-X1      TCATAATAAGAACATGCAACCAGAGTGGAGACAAATTTGTTGTAAGAGTTAGGATTTCTG 484
GmMKS2-X3      TCATAATAAGAACATGCAACCAGAGTGGAGACAAATTTGTTGTAAGAGTTAGGATTTCTG 600
                                      *************************************

GmMKS2-X2      GCTCTTCAGCTGCTCGTTTATACTTTGATCACTTCATCTATAAGCTGCCAAACCAAGAGC 499
GmMKS2-X1      GCTCTTCAGCTGCTCGTTTATACTTTGATCACTTCATCTATAAGCTGCCAAACCAAGAGC 544
GmMKS2-X3      GCTCTTCAGCTGCTCGTTTATACTTTGATCACTTCATCTATAAGCTGCCAAACCAAGAGC 660
               ************************************************************

GmMKS2-X2      CTATTTTGGAAGCCAAGGCCATAGCGGTGTGGCTTGACAAAAACTATCGTCCTATACGAA 559
GmMKS2-X1      CTATTTTGGAAGCCAAGGCCATAGCGGTGTGGCTTGACAAAAACTATCGTCCTATACGAA 604
GmMKS2-X3      CTATTTTGGAAGCCAAGGCCATAGCGGTGTGGCTTGACAAAAACTATCGTCCTATACGAA 720
               ************************************************************

GmMKS2-X2      TTCCAGCAGAGATGAAGTCTAAATTTGTAAAGTTTATTCGAATTGAGGACTCTTAA     615
GmMKS2-X1      TTCCAGCAGAGATGAAGTCTAAATTTGTAAAGTTTATTCGAATTGAGGACTCTTAA     660
GmMKS2-X3      TTCCAGCAGAGATGAAGTCTAAATTTGTAAAGTTTATTCGAATTGAGGACTCTTAA     776
               ********************************************************
```

(b)

```
GmMKS2-X2    1 MSYNHTSSMSLPSPLYLNTTSFRLTRQSPFPFPRRRFNPPAFRSVSPLSSSPSASLFDLR
GmMKS2-X1    1 MSYNHTSSMSLPSPLYLNTTSFRLTRQSPFPFPRRRFNPPAFRSVSPLSSSPSASLFDLR
GmMKS2-X3    1 MSYNHTSSMSLPSPLYLNTTSFRLTRQSPFPFPRRRFNPPAFRSVSPLSSSPSASLFDLR

GmMKS2-X2   61 GGKGMSGFHDVELKVRDYELDQYGVVNNAVYASYCQHGRHELLQNIGINCDAVARSGDAL
GmMKS2-X1   61 GGKGMSGFHDVELKVRDYELDQYGVVNNAVYASYCQHGRHELLQNIGINCDAVARSGDAL
GmMKS2-X3   61 GGKGMSGFHDVELKVRDYELDQYGVVNNAVYASYCQHGRHELLQNIGINCDAVARSGDAL

GmMKS2-X2  121 ALSELSLKFLAPLR---------------SGDKFVVRVRISGSSAARLYFDHFIYKLPNQ
GmMKS2-X1  121 ALSELSLKFLAPLRFCFPIFVLIIRTCNQSGDKFVVRVRISGSSAARLYFDHFIYKLPNQ
GmMKS2-X3  121 ALSELSLKFLAPLRV--------------SLSNFACHFCLLGYSAT--------------

GmMKS2-X2  166 EPILEAKAIAVWLDKNYRPIRIPAEMKSKFVKFIRIEDS
GmMKS2-X1  181 EPILEAKAIAVWLDKNYRPIRIPAEMKSKFVKFIRIEDS
GmMKS2-X3  153 ---VQITSLYILVH-------------------------
```
