# Supplementary material for: Identification and Functional Characterization of a Soybean (Glycine max) Thioesterase that Acts on Intermediates of Fatty Acid Biosynthesis
Source: Plants (Basel). 2019 Oct 8;8(10):397. doi: 10.3390/plants8100397 (PMC6843456; doi:10.3390/plants8100397)
Supplement: Supplementary file 1 [file plants-08-00397-s001.zip › Supplementary Files_proofed/Supplementary File 6_revised.pdf]

Supplementary File 6. Putative *cis*-regulatory elements in the GmMKS2 promoter identified through PLACE database [65-95].

Cis-acting elements	Sequence	Location (Strand)	Putative Function
AAAG motif	AAAG	-988 (-), -877 (+), -816 (+), -808 (+), -752 (-), -660 (-), -608 (-), -530 (-), -410 (+), -303 (-), -283 (-), -155 (-), -137 (+), -40 (+), -23 (+), +26 (+), +51 (+)	binding site of Dof proteins essential for multiple gene expressions associated with the plant-specific pathway for carbon metabolism [65,66]
T-box	AACGTT	-983 (+/-)	*cis*-acting regulatory element involved in the MeJA-responsiveness [42,67]
ACGT element	ACGT	-982 (+/-), -908 (+/-)	*cis*-acting element involved in plant responses to dehydration [49]
DPBF core	ACACNNG	-963 (+), -909 (-)	binding site of bZIP transcription factors involved in the seed-specific and ABA-responsive expression of *Dc3* gene [68]
E-box (MYC motif)	CANNTG	-962 (+/-), -930 (+/-), -919 (+/-), -909 (+/-), -901 (+/-), -793 (+/-), -627 (+/-), -480 (+/-)	MYC recognition site found in the promoters of the dehydration-responsive genes such as *rd22* [50,69], binding site of ICE1 that regulates the transcription of CBT/DREB1genes in the cold [70]
NGATT motif	NGATT	-958 (+), -803 (+), -473 (+), -359 (+), -239 (+), -146 (+), -88 (+), +51(+), +88 (-, +94(-)	*cis*-acting regulatory element involved in cytokinin responsiveness [71]
W-box core motif	(T)TGAC(C/T)	-951 (-), -866 (-), -444 (-), -413 (-)	binding site of WRKY transcription factors [72]
ARE	AAACAAA	-939 (+), -17 (+)	anaerobic responsive element [73]
Circadian	CAANNNNATC	-936 (+)	necessary for circadian expression [74]
GARE	TAACAAR	-922 (+)	gibberellin-responsive element [75]
G-box	CACGTG	-909 (+/-)	recognized by the GBF (G-box factors) family of bZIP proteins involved in light responsiveness [76,77]
ABRE-like sequence	ACG(T/C)G	-909 (-), -908 (+)	involved in ABA responsiveness and Ca^{2+} regulation [49,78]

ElRE (Box W1)	TTGACC	-867 (-), -414 (-)	elicitor-responsive element [39,41,72,79]
CARE (CAACTC regulatory elements)	CAACTC	-843 (-)	gibberellin-responsive element [80]
CAAT box	CAAT	- 820(+), -543 (-), -453 (-), -428 (-), -338 (+), -237 (-), -196 (+), - 86 (-)	common *cis*-acting element in promoter and enhancer regions, and essential for optimum transcriptional activity [81]
SRE	TTATCC	-812 (-)	sugar-repressive element (SRE) found in the dehydrin gene promoters and in the promoters of genes down-regulated after main stem decapitation [82]
GATA-box	GATA	-811 (+), -765 (+), -592 (-), -559 (+), -463 (+), -425 (+), -334 (+), -306 (-), -290 (+), -118 (+), -116 (-), -35 (+)	required for light regulated and tissue specific expression [83]
I-box	GATAA	-811 (+)	Light-responsive element [83]
DRE core motif	(A/G)(T/C)CG AC	-805 (-)	binding site for C-repeat binding factors (CBFs), also known as dehydration responsive element (DRE) binding proteins (DREBs) [84]
CAACA motif	CAACA	-789 (-), -476 (-), -457 (+), -448 (-), -214 (-), -106 (+)	AP-2 binding *motifs* [85]
L-box	ACC(A/T)AC C	-777 (-)	MYB-binding motif involved in response to elicitor treatment and UV-B irradiation [86]
$(CA)_n$ element	CNAACAC	-737 (-)	important for embryo- and endosperm-specific transcription [87]
SORLIP	GCCAC<br>CTCAAGTGA	-671 (-), -597 (+)<br>-628 (-)	Sequences Over-Represented in Light-Induced Promoters (SORLIPs) [77]
AG consensus	TTWCCWWW WNNGGWW	-664 (+)	binding consensus sequence for floral homeotic protein AGAMOUS (AG) [88]
RY repeat	CATGCA	-603 (-)	found in RY/G box and required for seed specific expression, a binding site for B3 domain transcription factors [87,89]

CCAAT box	CCAAT	-543 (-), -453 (-), -237 (-)	found in the promoter of certain heat shock protein genes and acts cooperatively with heat-shock elements to increase the promoter activity [90]
SEF3 motif	AACCCA	-541 (-), +71 (-)	binding site of soybean embryo factor 3 (SEF3) [91]
MYB-related motif	YAACKG<br>CNGTTR	-480 (-), +98 (-)<br>-480 (+), +98 (+)	binding site for MYB transcriptional activators involved water stress responsiveness [50,92]
WB box	TTTGACY	-414 (-)	binding site for WRKY proteins [72,79]
TATA box	TATAAAT<br>TATATAA	-188 (+)<br>-176 (-), -175 (+)	core promoter element required for RNA polymerase II to initiate transcription [93]
GT-1 motif	GAAAAA	-48 (+), +45 (+)	involved in pathogen- and salt-induced expression of *CaM-4* gene from soybean [94]
Pyrimidine box	TTTTTTCC	+44 (-)	required for GA induction [95]
