# Supplementary figures and images for: Identification and Functional Characterization of a Soybean (Glycine max) Thioesterase that Acts on Intermediates of Fatty Acid Biosynthesis
Source: Plants (Basel). 2019 Oct 8;8(10):397. doi: 10.3390/plants8100397 (PMC6843456; doi:10.3390/plants8100397)

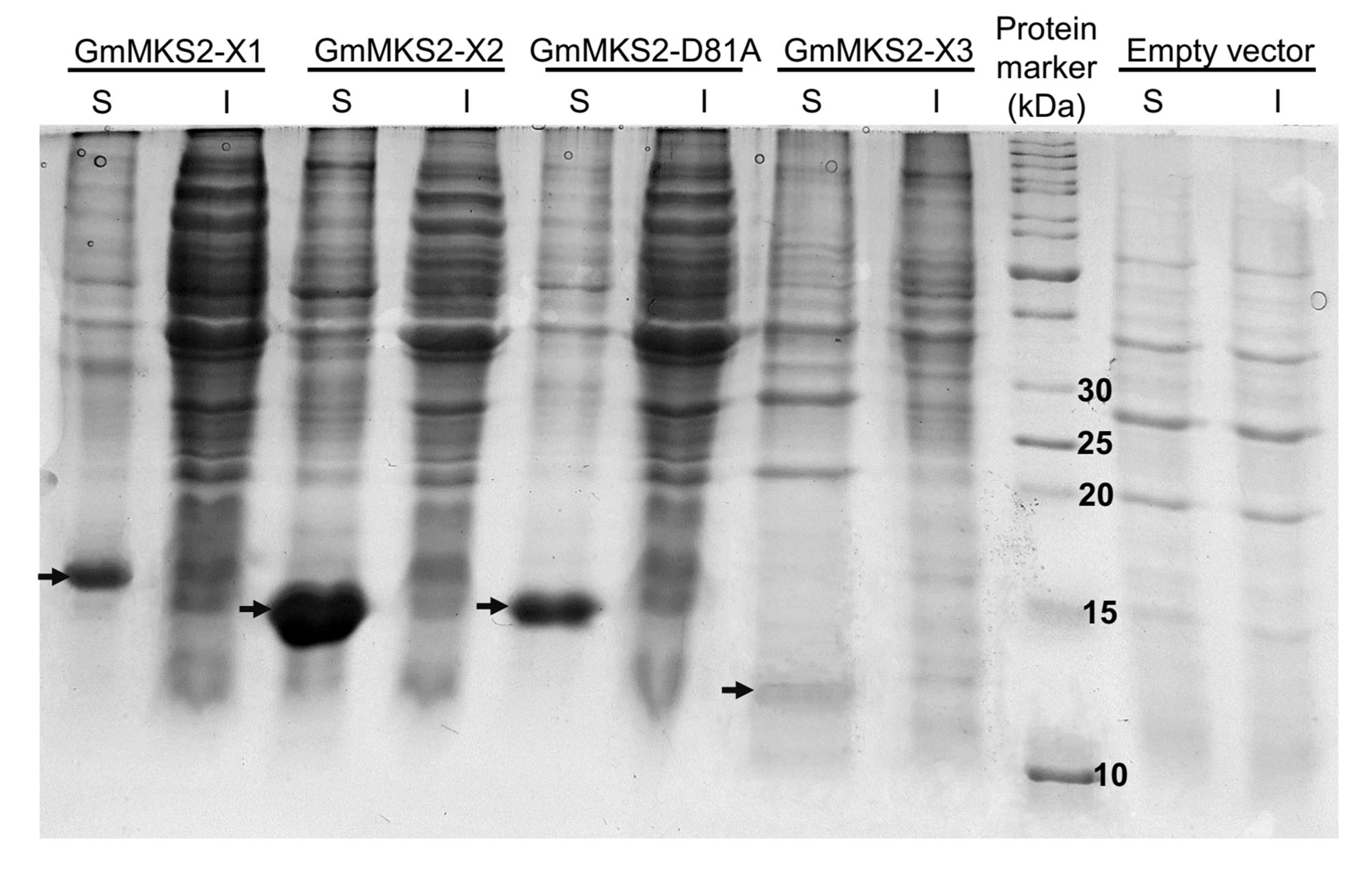

Supplement: Supplementary file 1 [file plants-08-00397-s001.zip › Supplementary Files_proofed/Supplementary File 4_revised.jpg]

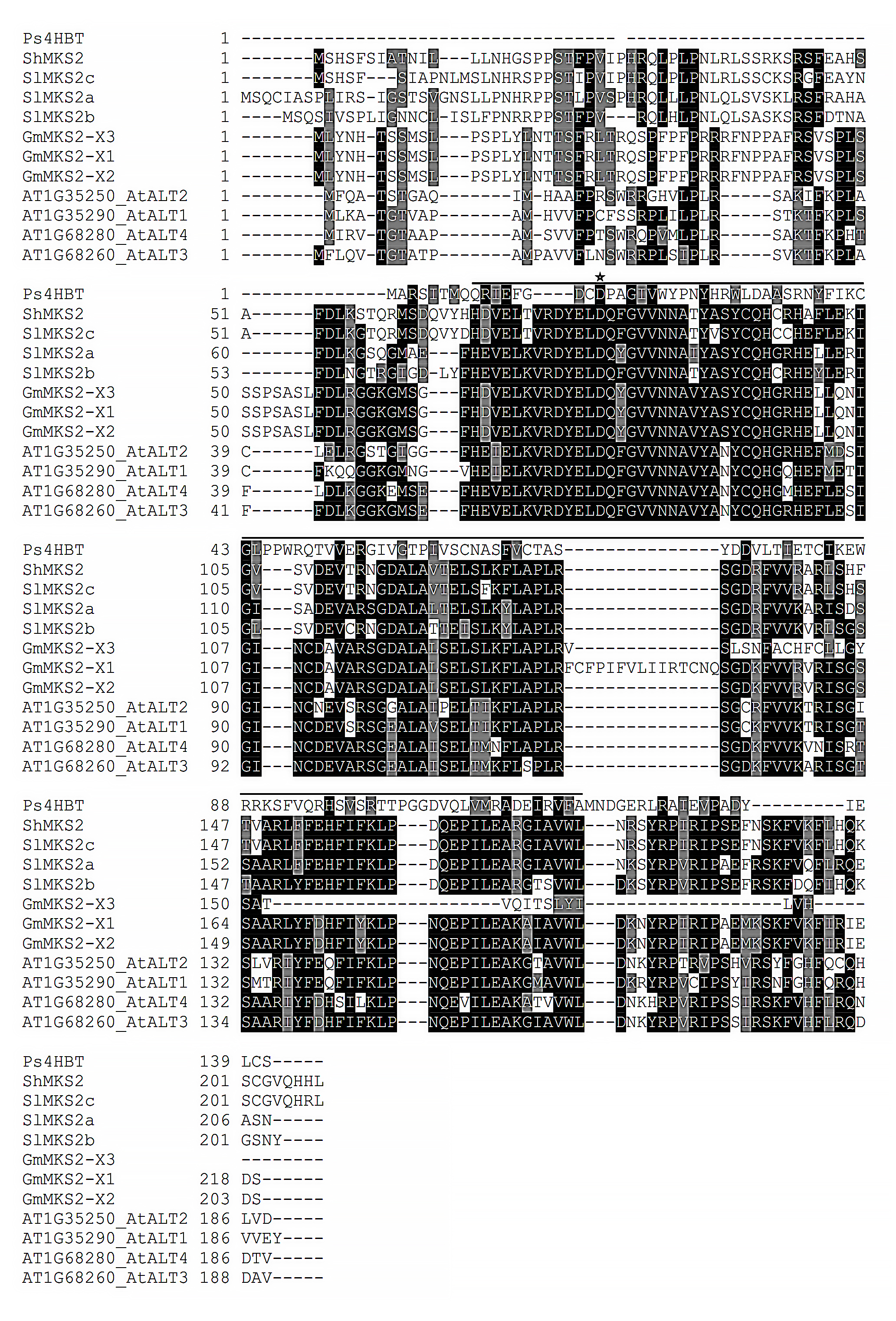

Supplement: Supplementary file 1 [file plants-08-00397-s001.zip › Supplementary Files_proofed/Supplementary File 5_revised.jpg]

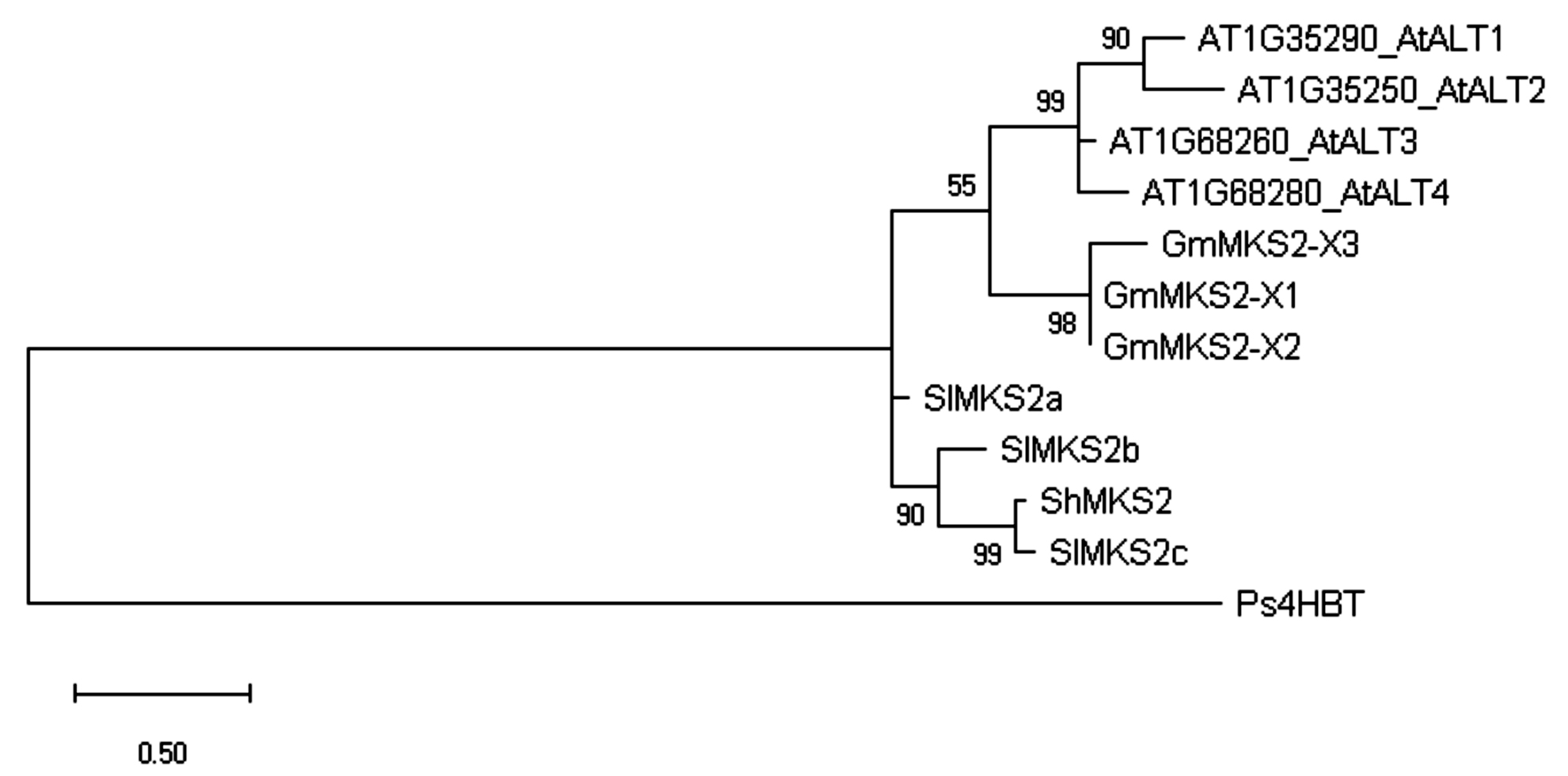

Supplement: Supplementary file 1 [file plants-08-00397-s001.zip › Supplementary Files_proofed/Supplementary File 7_revised.jpg]

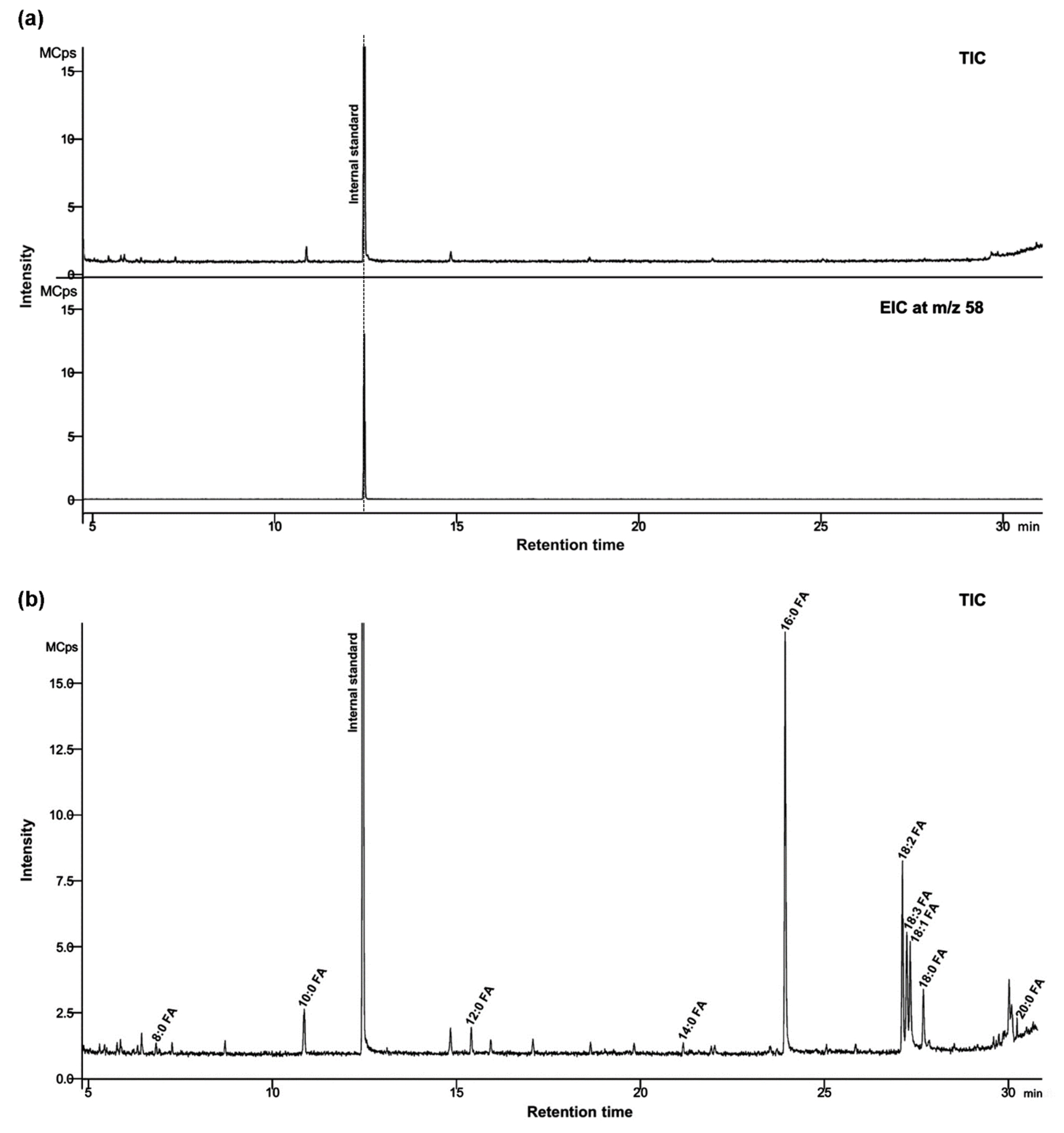

Supplement: Supplementary file 1 [file plants-08-00397-s001.zip › Supplementary Files_proofed/Supplementary File 8_revised.jpg]
